# Gate-tunable mid-infrared electroluminescence from Te/MoS_2_ p-n heterojunctions

**DOI:** 10.1038/s41377-026-02402-6

**Published:** 2026-07-27

**Authors:** Shiyu Wang, Delang Liang, Zhi Zheng, Mingyang Qin, Yuchun Chen, Jie Sheng, Shula Chen, Lin Li, Changgan Zeng, Anlian Pan, Jinluo Cheng, Dong Sun

**Affiliations:** 1 International Center for Quantum Materials, School of Physics, Peking University, Beijing, China; 2 Key Laboratory for Micro-Nano Physics and Technology of Hunan Province, Hunan Institute of Optoelectronic Integration, College of Materials Science and Engineering, Hunan University, Changsha, China; 3 CAS Key Laboratory of Strongly Coupled Quantum Matter Physics, and Department of Physics, University of Science and Technology of China, Hefei, China; 4 School of Physics and Electronics, Hunan Normal University, Changsha, China; 5 School of Physics and Laboratory of Zhongyuan Light, Zhengzhou University, Zhengzhou, China; 6 GPL Photonics Laboratory, State Key Laboratory of Luminescence Science and Applications, Changchun Institute of Optics Fine Mechanics and Physics, Chinese Academy of Sciences, Changchun, China; 7 Collaborative Innovation Center of Quantum Matter, Beijing, China; 8 Frontiers Science Center for Nano-optoelectronics, School of Physics, Peking University, Beijing, China

**Keywords:** Lasers, LEDs and light sources, Mid-infrared photonics, Electronics, photonics and device physics

## Abstract

Mid-infrared (MIR) emitters are critical components in advanced photonic systems, driving progress in fields such as chemical sensing, environmental monitoring, medical diagnostics, thermal imaging and free-space communications. Conventional MIR emitters based on III–V heterostructures rely on complex epitaxial growth on rigid lattice-matched substrates and suffer from limited integration compatibility with CMOS or flexible platforms. Recent MIR emitters based on two-dimensional (2D) materials such as black phosphorus (BP) is more suitable for on-chip applications but faces challenges related to stability and emission efficiency. Based on the recently discovered highly efficient photoluminescence of Te, we demonstrate a gate-tunable mid-infrared light-emitting diode based on a van der Waals heterojunction formed by multilayer transition metal dichalcogenide (TMD) MoS_2_ and tellurium (Te). The device emits polarized electroluminescence (EL) centered at 3.5 μm under forward bias at 25 K, and the EL persists up to 80 K with reduced intensity. Gate control of the MoS_2_ Fermi level modulates the band alignment and injection efficiency, enabling dynamic tuning of the EL intensity. The emission remains spectrally stable under varying bias and gating, indicating robust band-edge recombination. These results establish the Te/TMD heterostructure as a promising platform for integrated polarized mid-infrared optoelectronics.

## Abstract (web summary)

Gate-controlled band engineering in Te/MoS_2_ heterojunctions enables wavelength-stable, linearly polarized and electrically tunable emission toward on-chip mid-infrared polarized light sources.

## Graphical abstract

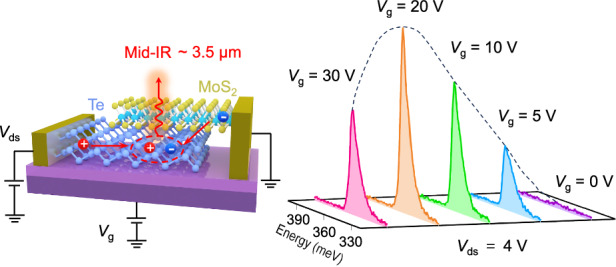

## Introduction

The mid-infrared (MIR, 2.5–25 μm) spectral region is essential for numerous applications, including gas sensing, infrared spectroscopy, thermal imaging, and free-space optical communication^1–3^. Conventional MIR light-emitting technologies have been developed primarily on epitaxial III–V platforms, including InSb/AlInSb quantum-well light-emitting diodes^4^, GaInAsSbP alloy light-emitting diodes^5^, and intersubband devices based on III–V compounds^6,7^. Within the same epitaxial technology landscape, type-II InAs/GaSb superlattice interband LEDs established cascaded active-region concepts for 3–4 μm electroluminescence^8^ and were subsequently advanced toward higher radiance and output power through optimized cascaded designs, including work from John P. Prineas and co-workers^9^. Interband cascade LEDs further pushed continuous-wave mid-IR LED power into the multi-milliwatt regime, with notable progress from industrial development efforts such as nanoplus^10^. More recently, mercury-chalcogenide colloidal quantum-dot LEDs, including HgTe and HgSe-based systems from Philippe Guyot-Sionnest’s group, have emerged as a solution-processable route to mid-IR electroluminescence^11,12^. Despite their strong performance, these established approaches typically rely on epitaxial growth on lattice-matched substrates and device stacks that are less compatible with CMOS back-end processing or mechanically flexible platforms. Moreover, polarization-defined emission and spectral engineering in many mid-IR emitters are commonly implemented by incorporating additional photonic structures such as resonant cavities or gratings, while the growing demand for on-chip mid-IR polarimetry motivates compact sources with intrinsic and electrically controllable polarization. These constraints have spurred interest in alternative optoelectronic systems that combine mechanical flexibility, process scalability, and spectral tunability. Two-dimensional (2D) van der Waals (vdW) semiconductors offer distinct advantages for light emitters^13–19^, including atomic thinness, electrostatic tunability, and seamless integration on arbitrary substrates without lattice-matching constraints. Following the isolation of monolayer transition metal dichalcogenides (TMDs), electroluminescence (EL) was rapidly demonstrated in monolayer MoS_2_^14^ and WSe₂, whose direct bandgaps and strong excitonic effects enable bright emission in the visible and near-infrared (NIR) regimes. Various device architectures—such as lateral p-n junctions^20^, vertical heterostructures^15^, and tunneling diodes^16–18^—have since been developed to realize valley polarization-tunable and gate-controlled EL^19^ based on TMDs. Despite these advances, the relatively large bandgaps of TMDs (>1 eV) fundamentally limit their operation wavelength range to the visible-NIR region, thereby limiting their applicability in the mid-infrared region. In addition, light emission from TMDs is not polarized due to the nearly isotropic in-plane optical response of TMDs, and it typically requires external photonic structures to achieve strong linear polarization^21^. This is in contrast to intrinsically anisotropic 2D semiconductors such as black phosphorus or Te. As a result, TMD-based emitters are not ideal for applications in the mid-infrared region.

**Fig. 1:**
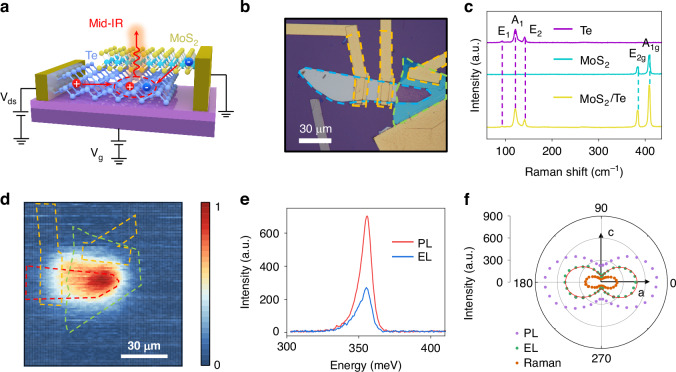
Schematic and optical properties of the Te/MoS_2_ heterojunction. **a** Schematic illustration of the Te/MoS_2_ heterojunction device. **b** Optical micrograph showing the Te/MoS_2_ heterojunction region (outlined by blue/green dashed lines) on a Si/SiO₂ (285 nm) substrate used as the back-gate. Scale bar: 30 μm. **c** Raman spectra of isolated Te (purple), MoS_2_ (blue), and the heterojunction region (yellow). **d** Spatially resolved EL intensity map of the Te/MoS_2_ heterojunction LED, recorded in the 3.5 µm band under forward bias (${V}_{{\rm{g}}}=20\,{\rm{V}},{V}_{{\rm{ds}}}=4\,{\rm{V}};25\,{\rm{K}}$). The Te flake and MoS_2_ region are outlined by red and green dashed lines, respectively. **e** PL (red) and EL (blue) spectra of the Te/MoS_2_ diode. **f** Polarization characterization of PL, EL, and the A₁ Raman mode. All curves are fit by $I=\left({I}_{\max }-{I}_{\min }\right){\cos }^{2}\theta +{I}_{\min }$, where *θ* is measured from the a-axis of Te, and *I*_max_ and *I*_min_ denote the intensities along the a-axis and c-axis for each polar plot, respectively. The crystal orientations are labeled with black arrows

To overcome these challenges, there has been growing interest in anisotropic 2D semiconductors with narrower bandgaps and polarization-selective optical properties. Black phosphorus (BP) has emerged as a promising MIR emitter, with a thickness-dependent direct bandgap ranging from ~0.3 eV (bulk) to ~2.0 eV (monolayer)^22–24^ and pronounced in-plane anisotropy that enables linearly polarized emission^22,25–27^. BP-based MIR light-emitting diodes have been investigated across diverse architectures, including heterojunction emitters, waveguide-integrated implementations, resonant-cavity designs, and van der Waals heterostructures, with recent progress toward scalable device formats^26–30^. A major impediment, however, is BP’s limited ambient stability^31,32^, which necessitates effective encapsulation for reliable operation. Encouragingly, long operating lifetimes have been demonstrated for properly encapsulated BP LEDs, highlighting the practical importance of stringent environmental isolation for BP-based MIR emitters. Moreover, BP exhibits strong electric field-induced spectral tunability via the Stark effect—an attractive route for active wavelength control, yet one that can also introduce emission-energy shifts under vertical electric fields^33^. Recently, tellurium (Te)^34–37^, a layered narrow-bandgap (~0.35 eV) p-type semiconductor, has attracted growing attention as an alternative to BP for MIR optoelectronics. Te possesses a thickness-independent bandgap that is nominally indirect with a small k-offset but effectively quasi-direct for optical transitions, along with excellent air stability, high mobility^38,39^, and intrinsic linear dichroism^40^. Prior studies have demonstrated MIR photodetection^40–42^ and highly efficient photoluminescence^43^ in Te-based heterostructures, highlighting its potential for MIR optoelectronics. Very recently, during the preparation of this work, electrically driven mid-infrared electroluminescence has been demonstrated in Te-based van der Waals LEDs that rely on dual-electrode bipolar injection, and the polarization of the emission can be tuned by bias^44^. These advances also link Te-based MIR emitters to Weyl physics, opening research avenues beyond the scope of conventional band-edge optoelectronics^45,46^. In this work, we develop a gate-tunable heterojunction of Te that enables systematic modulation of the built-in junction electrostatics and interfacial band alignment to control injection and recombination. Based on a vdW p-n heterojunction formed by n-type multilayer MoS_2_ and p-type Te, as schematically illustrated in Fig. 1a, we demonstrate a mid-infrared light-emitting diode. The band diagram highlights carrier injection and radiative recombination processes localized within Te, giving rise to EL emission centered at 3.5 μm. Gate-tunable carrier modulation enables control over the band alignment and interfacial injection efficiency. The device exhibits linearly polarized EL under forward bias, with the lowest turn-on voltage of ~1.7 V. Spectral analyses confirm that EL arises from direct band-edge transitions in Te, consistent with its PL response. Importantly, the Te/MoS_2_ platform demonstrates wavelength-stable and polarization-locked MIR emission under electrostatic control, and we further verify its operational robustness through extended storage (~10 months) without the need for strict hermetic encapsulation. These findings establish the Te/MoS_2_ heterostructure as a promising platform for gate-tunable, polarized mid-infrared light sources in reconfigurable photonic and optoelectronic circuits.

## Results

### Characterization of transport and optical properties

The device was fabricated via standard mechanical exfoliation and van der Waals (vdW) assembly processes, where multilayer MoS_2_ was positioned on top of a Te nanosheet. The source and drain electrodes were patterned by electron beam evaporation of Pd/Au (20 nm/100 nm) and Cr/Au (20 nm/100 nm) onto the Te flake and MoS_2_ layers, respectively. The 285 nm SiO₂ layer serves as the back-gate dielectric, with the p-type silicon substrate used as the back gate. Figure 1b shows an optical micrograph of a typical Te/MoS_2_ heterostructure, where the boundaries of the MoS_2_ and Te nanosheets are outlined by dashed blue and green lines, respectively. The Au electrodes are visible as bright metallic regions, outlined by orange dashed lines. To determine the thickness and surface morphology of the MoS_2_/Te heterojunction, atomic force microscopy (AFM) measurements were performed over the sample area. The thicknesses of MoS_2_ and Te were measured to be ~17 and 234 nm, respectively (see Supplementary Information Fig. S1). Raman spectroscopy was performed to confirm the composition and crystalline quality of the heterostructure. For Raman measurement, a 532 nm laser was utilized to excite the characteristic resonant peaks in Te, MoS_2_, and the heterojunction region. As shown in Fig. 1c, the Te-related peaks at 92, 120, and 140 cm^−1^ are assigned to the E_1_ (bond-bending mode), A (chain expansion mode), and E_2_ (bond-stretching mode) phonon vibrational modes; the MoS_2_-related peaks at 385 and 409 cm^−1^ are assigned to the E_2g_ (in-plane Mo-S vibration mode) and A_1g_ (out-of-plane mode), respectively. In the heterostructure region, all peaks retain their spectral positions without broadening or shifting, demonstrating negligible interlayer strain and preserved lattice integrity at the interface. No additional Raman modes are observed in the overlap region, consistent with a structurally intact van der Waals heterojunction.

We focused on characterizing the device’s EL emission under source-drain bias first. To gain deeper insight into the origin of this EL emission, we also carried out photoluminescence (PL) measurements of the Te/MoS_2_ heterostructure under 1064 nm laser excitation. As shown in Fig. 1e, the resulting PL and EL spectra are presented together for direct comparison. Under forward-bias conditions (*V*_g_ = 20 V, *V*_ds_ = 4 V), the device exhibits EL emission centered at ~3.5 μm. All measurements were performed at 25 K, unless otherwise noted, to enable high signal-to-noise detection of gate-modulated mid-infrared emission. As shown in Supplementary Note 4, electroluminescence persists up to 80 K, although the emission intensity decreases with increasing temperature. A direct spectral comparison with PL reveals that both EL and PL exhibit nearly identical peak positions and spectral linewidths (Fig. 1e), suggesting that both emissions originate from interband transitions of Te, a quasi-direct transition between the valence band maximum and conduction band minimum of Te. Spatially resolved EL mapping (Fig. 1d) shows that the mid-infrared emission primarily originates from the Te/MoS_2_ overlap (junction) region. The device geometry in Fig. 1d was determined by comparing the EL map to a reflection micrograph of the same area, which is provided in Supplementary Fig. S5.

Given the in-plane anisotropic chain-like crystal structure of Te, the EL emission from Te-based heterostructures is expected to be strongly linearly polarized, similar to the polarized photoluminescence (PL) reported in our recent work^47^. The polarized emission is crucial for polarization-sensitive optoelectronic applications. Figure 1f presents measurements of the polarization characteristics of the EL emission. Notably, both EL and PL emissions reach maximum intensity when the polarization is aligned along the armchair direction (a-axis) and minimum intensity when the polarization is aligned along the chain direction (c-axis), reflecting the intrinsic optical anisotropy dictated by selection rules of the dipole matrix elements near the Te bandgap, where radiative transitions are preferentially oriented along the a-axis. To quantify the polarization, we use the degree of linear polarization (DOP), defined as $\mathrm{DOP}=\left({I}_{\max }-{I}_{\min }\right)/\left({I}_{\max }+{I}_{\min }\right)$. The DOP of the measured device is also bias dependent, consistent with a recent report on graphene-Te-graphene mid-infrared LEDs^44^. The EL polarization plot shown in Fig. 1f yields a DOP of 0.70. We note that the polarization state varies significantly across different devices. Different Te flakes in Te/TMD heterojunctions exhibit different degrees of linear polarization in EL, while in an independent Te/WSe_2_ device, we observe a nearly unity EL DOP (see Supplementary Fig. S6). The complex DOP results may arise from the flake-to-flake variations in the intrinsic doping of Te, which shifts the equilibrium Fermi level and thereby influences the polarization; and recent Te-based MIR LEDs have shown that the DOP can vary with the injection condition^44^. Local strain and substrate-induced effects are also possible contributing factors. A comprehensive bias‑ and gate‑dependent study of the polarization behavior is beyond the scope of this work and requires further investigation. Having established that the Te/MoS_2_ heterostructure functions as a polarized mid‑infrared light emitter, we next examine its electronic transport properties and interfacial band alignment. In the present device platform, Te flakes were selected from hydrothermal batches to ensure sufficient morphological uniformity for reliable junction formation, while few-layer MoS_2_ was employed to provide robust electron transport and stable current drive for reproducible EL turn-on. A brief rationale for the thickness selection is provided in Supplementary Note 1.

**Fig. 2:**
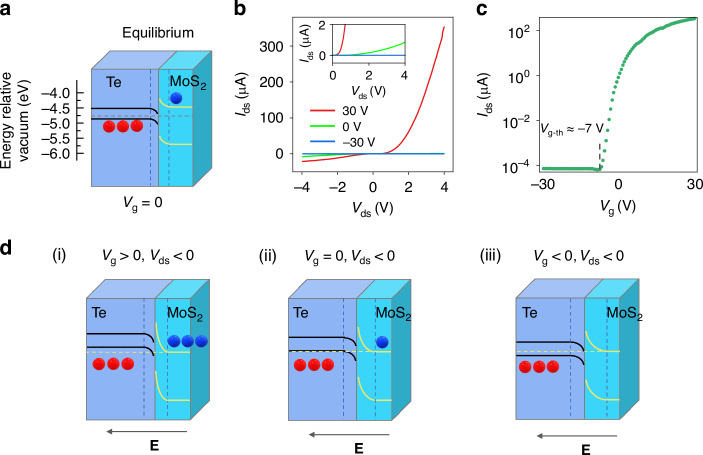
Band alignment and electrical transport of the Te/MoS_2_ heterojunction. **a** Qualitative equilibrium band alignment of the Te/MoS_2_ heterojunction at *V*_g_ = 0 V, with the depletion region marked by gray dashed lines. **b** Output characteristics (*I*_ds_-*V*_ds)_ of the heterostructure measured at 25 K for back-gate voltages *V*_g_ = 30, 0, and −30 V, showing gate-tunable rectification behavior. The inset provides a magnified view of the low-current region. **c** Transfer characteristics (*I*_ds_-*V*_g_) recorded at *V*_ds_ = 4 V. **d** Schematic band alignment of the Te/MoS_2_ heterojunction under reverse bias (*V*_ds_ < 0) for three representative gate voltages: (i) *V*_g_ > 0, (ii) *V*_g_ = 0, and (iii) *V*_g_ < 0. The yellow dashed line indicates the position of the MoS_2_ conduction band edge

The electrical properties of the Te/MoS_2_ heterostructure were systematically investigated to elucidate its p-n junction behavior and gate-dependent transport characteristics at 25 K. The output characteristics (*I*_ds_-*V*_ds_) measured at back-gate voltages (*V*_g_) of 30, 0, and –30 V show pronounced, gate-tunable rectification behavior, as depicted in Fig. 2. At *V*_g_ = +30 V, when the MoS_2_ channel is strongly n-doped, the device exhibits clear rectification behavior. At *V*_g_ = 0 V, the rectification is strongly suppressed, and the currents remain in the submicroampere range over the entire *V*_ds_ window. At *V*_g_ = –30 V, the current stays at the measurement noise floor over the *V*_d*s*_ range, indicating that carrier injection across the junction is effectively quenched by gate-induced depletion.

We first recall the equilibrium band alignment and then consider how the applied gate voltage and bias voltage modify carrier injection at the junction. Figure 2a shows a schematic of the equilibrium band alignment in the Te/MoS_2_ heterojunction at zero gate voltage. In this configuration, the p-type Te and n-type MoS_2_ result in hole accumulation in Te and electron accumulation in MoS_2_. Hall measurements on representative Te flakes exhibit a typical natural hole doping on the order of 5 × 10^18 ^cm^−3^ (ref. ^48^). At the interface, the redistribution of free carriers forms a depletion region (indicated by gray dashed lines), where band bending gives rise to a built-in electric field. Previous reports determined the Te and MoS_2_ work functions (*Φ*_Te_ ≈ 4.75 eV and *Φ*_MoS2_ ≈ 4.3 eV) by UPS/KPFM^41,49^. Accordingly, the band diagram in Fig. 2a is constructed using these quantitative data and provides a quantitative basis for the equilibrium band alignment. Importantly, the large valence band offset at the Te/MoS_2_ interface serves as an effective energy barrier, preventing hole leakage from Te into MoS_2_.

To rationalize the gate‑dependent band diagrams in Fig. 2d, it is important to consider how the back gate voltage couples to the Te and MoS_2_ layers in this vertical geometry. The Te/MoS_2_ heterojunction and the back silicon electrode form a capacitor. Because the MoS_2_ layer is relatively thin and lies directly on the gate dielectric outside Te, the back-gate voltage primarily modulates the carrier density in the edge regions of MoS_2_ rather than in the region where MoS_2_ overlies Te. In this geometry, the gate field effectively penetrates the full thickness of the ~17 nm MoS_2_ channel and tunes its Fermi level, while the much thicker Te is strongly screened and remains only weakly affected by the back gate. This diagram of the gating effect is consistent with the EL spatial map shown in Fig. 1d. The EL-active region extends across the Te/MoS_2_ overlap area. Although the back gate couples most efficiently to the MoS_2_ regions that are not covered by Te, carriers can be efficiently injected into the whole overlap area with Te, which contributes to the EL emission from the overlapped area. Consequently, back-gating provides an efficient handle to tune the effective band alignment and injection across the Te/MoS_2_ interface and thus the EL output from the overlap region.

For *V*_g_ = 0 V and +30 V, a finite reverse-bias current persists at sufficiently negative *V*_ds_, which can be attributed to band-to-band tunneling. As illustrated in Fig. 2d(i, ii), the combined effect of the drain-source bias *V*_ds_ and the back-gate voltage *V*_g_ bends the bands such that the conduction band minimum of MoS_2_ lies below the valence band maximum of Te, resulting in a type-II band configuration. When the magnitudes of *V*_ds_ are fixed in Fig. 2d, positive *V*_g_ shifts the MoS_2_ conduction band downward in energy relative to Te and increases the overlap between the MoS_2_ conduction band and Te valence band. This effect enhances the tunneling probability and produces a larger reverse current at *V*_g_ > 0 than at *V*_g_ = 0, which is consistent with the transport data shown in Fig. 2b. In contrast, for *V*_g_ < 0 (Fig. 2d(iii)), the MoS_2_ conduction band is shifted upward in energy relative to the Te valence-band edge, so the band-to-band tunneling window effectively closes, and the reverse current is strongly suppressed. The transfer characteristics (*I*_ds_-*V*_g_) measured at *V*_ds_ = 4 V confirm that the device exhibits pronounced rectifying behavior, with the current strongly dependent on gate-induced carrier modulation. The threshold voltage (*V*_th_) of the MoS_2_ FET was determined to be ~−7 V. This value was extracted from the logarithmic *I*_ds_-*V*_g_ transfer curve (Fig. 2c) as the gate voltage at which the drain current departs from the subthreshold regime and begins to increase rapidly. These results demonstrate that the Te/MoS_2_ heterostructure operates as a robust and gate-tunable p-n junction, with rectifying behavior and suppressed leakage arising from the built-in potential at the interface. These characteristics provide a consistent electronic framework for understanding the bias‑ and gate‑dependent electroluminescence discussed later.

### Mid-infrared electroluminescence and bias-dependent spectra of Te/MoS_2_ heterojunctions

**Fig. 3:**
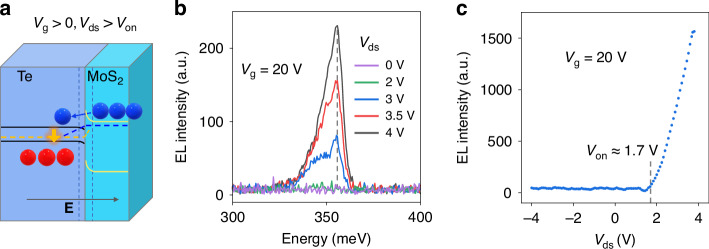
Band alignment and bias-dependent electroluminescence of the Te/MoS_2_ heterojunction. **a** *V*_ds_ > *V*_on_ (*V*_g_ = 20 V), illustrating the light-emission process. Blue and orange dashed lines denote quasi-Fermi levels for electrons and holes, respectively. **b** Bias-dependent EL spectra at *V*_g_ = 20 V (25 K). **c** Integrated EL intensity as a function of bias voltage

We next discuss the electroluminescence and underlying recombination mechanisms under different bias conditions. To directly correlate this band‑alignment diagram with light emission, we investigate how the mid‑infrared EL depends on the applied source-drain bias at a fixed gate voltage. Bias-dependent EL spectra (Fig. 3b) are obtained by sweeping the source-drain voltage (*V*_ds_) from −4 to 4 V while keeping the gate voltage fixed at *V*_g_ = 20 V. EL emission is observed only when the forward bias exceeds an EL turn-on voltage (*V*_on_). We define *V*_on_ as the minimum *V*_ds_ at which the integrated EL intensity clearly exceeds the background signal. *V*_on_ is not a fixed constant but varies systematically with gate voltage. At *V*_g_ = 20 V, the spectra shown in Fig. 3b indicate that *V*_on_ > 2 V and *V*_on_ is accurately determined to be below 1.7 V using the higher sensitivity measurement described in Supplementary Note 7. Notably, the optical turn-on voltage (*V*_on_ ~ 1.7 V) is larger than both the Te bandgap scale (*E*_g_/*q* ~ 0.35 V) and the estimated built-in potential (~0.46 V). This is expected because *V*_on_ reflects the bias required to reach sufficient interlayer injection and quasi-Fermi level splitting in the overlap region for detectable radiative recombination, together with additional voltage drops from contact/access series resistance. Near the onset, trap filling and defect-assisted nonradiative recombination can further delay the emergence of EL to larger voltage. For *V*_ds_ above *V*_on_, the EL intensity increases steadily when the applied bias increases further. The EL peak position remains essentially unchanged under different carrier injection levels, indicating a stable underlying radiative transition. The linewidth shows a weak narrowing trend with increasing bias (Supplementary Note 12). As the emission becomes increasingly dominated by band-edge recombination, the relative contribution from the low-energy-side emission decreases, leading to a slight reduction in the full width at half maximum. The low-energy in-gap emission may originate from shallow impurity states, the Franz-Keldysh effect induced by the built-in electric field, and excitonic emission. Figure 3a illustrates the band alignment under forward bias: the applied voltage flattens the electron barrier on the MoS_2_ side and thereby lowers the effective electron injection barrier. Under these conditions, the lowered and narrowed interfacial barrier favors tunneling-assisted electron injection from MoS_2_ into Te. At 25 K, the thermal energy (k_B_*T* ≈ 2.15 meV) is much smaller than the tens of meV barrier scale, and no obvious high-energy spectral broadening is observed, indicating that thermally activated over-barrier injection is not the dominant mechanism. At the same time, the large valence band offset at the Te/MoS_2_ interface maintains a high barrier for holes in Te. These two conditions together ensure that radiative recombination is spatially localized within the Te layer, which is responsible for the observed mid-infrared emission. The spatial profiles of the quasi-Fermi levels under forward bias lend further support to this interpretation. Specifically, the electron quasi-Fermi level in MoS_2_ exhibits a gradual downward slope as it approaches the interface, confirming efficient electron injection into Te. In contrast, the hole quasi-Fermi level in Te rises steeply near the boundary, forming a sharp gradient that signifies strong hole confinement and suppressed injection into MoS_2_. Under reverse bias, as shown in Fig. 2d(i), the applied voltage increases the built-in potential, thereby steepening the quasi-Fermi level gradients on both sides of the heterojunction. This enhancement strengthens carrier separation within the depletion region and effectively suppresses both electron and hole injection. As a result, radiative recombination is inhibited, leading to suppressed electroluminescence under reverse bias. These quasi-Fermi level profiles qualitatively explain the bias-dependent switch of EL emission. Long‑term operational stability data, showing that the device maintains robust mid‑infrared electroluminescence after extended ambient storage without any encapsulation, are provided in Supplementary Fig. S8.

### Gate voltage tunable electroluminescence of Te/MoS_2_

**Fig. 4:**
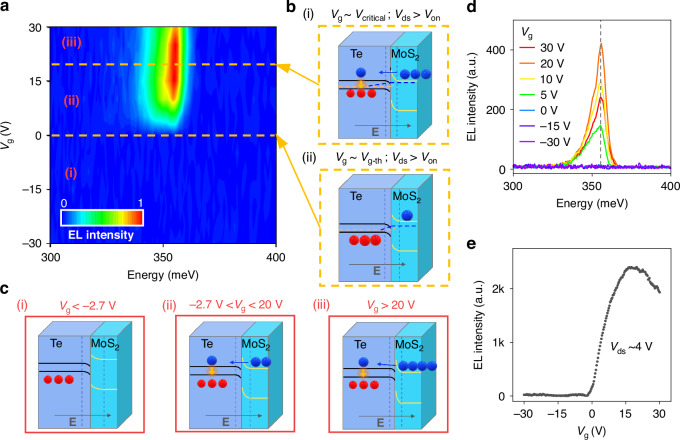
Gate voltage dependence of Te/MoS_2_ electroluminescence. **a** Plot of EL intensity as a function of photon energy and gate voltage *V*_g_ under a 4 V bias voltage. **b** Interfacial energy band alignment diagram of Te/MoS_2_ under critical gate voltages and threshold gate voltages, where diagrams in (i) and (ii) correspond to the energy bands of the yellow dashed lines in Fig. 4a. The blue and orange dashed lines in (i) and (ii) indicate the quasi-Fermi levels for electrons and holes, respectively. **c** Interfacial energy band alignment of Te/MoS_2_ across different gate voltage regions: (i) nonradiative states under *V*_g_ < −2.7 V, (ii) intermediate regime where *V*_g_ < *V*_g-crit_, and (iii) radiative bands at *V*_g_ > *V*_g-crit_. **d** Selected EL spectra at different gate voltages, ranging from 30 to −30 V, measured at 25 K. **e** Integrated intensity of the EL emission as a function of gate voltage (with fixed *V*_ds_ = 4 V), measured at 25 K

Having identified the bias window over which efficient mid‑infrared EL occurs, we next explored how electrostatic gating of the MoS_2_ channel modulates the EL intensity at fixed forward bias. Since EL emission in the Te/MoS_2_ heterostructure arises from radiative recombination between electrons injected from MoS_2_ and holes localized in Te, the EL intensity is highly sensitive to the doping level of MoS_2_. In particular, gate tuning of the doping level of MoS_2_ modulates the interfacial band alignment, thereby influencing the efficiency of carrier injection into the Te layer and ultimately tuning the light emission intensity. Since the back gate has little effect on the quasi-ohmic Te/Pd contacts (Fig. S2), its modulation of the doping level in MoS_2_ can slightly affect the built-in potential of MoS_2_/Cr contact, and further modulates the effective voltage applied on the MoS_2_/Te heterojunction. However, because the Schottky barrier of MoS_2_/Cr contact is only tens of meV, the effect of the built-in potential changes by the doping is expected to be limited, providing a minor correction compared with the heterojunction band alignment that governs the gate-tunable EL and is therefore omitted from the schematic band diagrams for clarity^50^. To elucidate this effect, we measured the EL response as a function of back-gate voltage *V*_g_ at *V*_ds_ = 4 V. Figure 4a displays the EL emission-energy map under a fixed lateral bias (*V*_ds_ = 4 V), revealing a nonmonotonic intensity profile: the EL emission shows a maximum at a critical gate voltage (*V*_g-crit_) of ~20 V.

This trend results from competition between enhanced electron accumulation and the formation of a gate-induced effective interfacial injection barrier. The underlying mechanism is illustrated by the band diagrams in Fig. 4b, c. Specifically, Fig. 4b highlights two representative gating conditions that define the onset and maximum of EL emission, while Fig. 4c traces the full evolution of the equilibrium band structure across the entire gating range. Figure 4b(i) corresponds to the critical gate voltage (*V*_g-crit_ = 20 V), where the EL emission is maximized because enhanced electron accumulation in MoS_2_ improves the electron supply while the gate-induced interfacial injection barrier increases. The competition between these two effects reaches an optimum balance at *V*_g-crit_. The quasi-Fermi-level profile in Fig. 4b(i) indicates that the MoS_2_ channel resistance is reduced at this gate bias due to a high carrier density, so that under the same applied *V*_ds_, a larger fraction of the voltage can drop across the junction region. Consistently, the electron quasi-Fermi level in MoS_2_ varies more gently toward the interface, which reflects a smaller series voltage drop in the MoS_2_ access region and thus more favorable injection conditions into Te. Meanwhile, the large valence-band offset at the Te/MoS_2_ interface blocks hole injection from Te into MoS_2_. Consistently, the hole quasi-Fermi level on the Te side varies sharply near the interface, indicating strong hole confinement. Figure 4b(ii) represents the threshold voltage (*V*_g-th_ = 0 V), where the gate-induced electron population in MoS_2_ first becomes sufficient to enable injection into Te, thereby lowering the effective electron injection barrier. In this case, the electron quasi-Fermi level exhibits a steeper spatial variation toward the junction, consistent with a larger series drop in MoS_2_ and injection that is only just initiated.

Figure 4c illustrates the full evolution of the band alignment in the Te/MoS_2_ heterostructure, complementing the specific gating conditions in Fig. 4b to offer a more comprehensive view. Three distinct gate-controlled regimes are identified:

(i) *V*_g_ < *V*_g-th_, Fig. 4c(i), (the depleted regime): below the threshold (*V*_g-th_), MoS_2_ is depleted of free electrons. The lack of available carriers quenches EL emission.

(ii) *V*_g-th_ < *V*_g_ < *V*_g-crit_, Fig. 4c(ii), (intermediate regime): as *V*_g_ increases above *V*_g-th_, electron accumulation in MoS_2_ increases, improving the electron supply for injection. The gate reshapes the electrostatic band bending near the interface and can increase the effective electron injection barrier. In this gate range, the benefit from increased electron supply dominates over the barrier penalty, so the net electron injection into Te and the EL intensity still increase with *V*_g_ and reach a maximum at *V*_g-crit_.

(iii) *V*_g_ > *V*_g-crit_, Fig. 4c(iii), (above 20 V, the high gate voltage regime): a higher gate voltage reshapes the electrostatic potential and band bending near the interface. This increases the effective electron injection barrier for carriers entering Te. The increased barrier suppresses electron injection into Te and becomes the dominant factor. It outweighs the benefit from additional electron accumulation, so the EL intensity no longer increases and decreases beyond *V*_g-crit_.

**Fig. 5:**
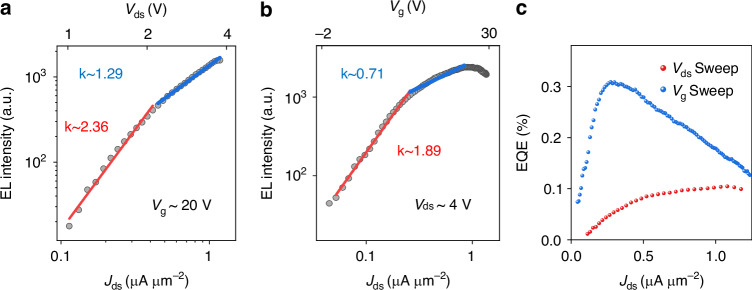
Current-dependent electroluminescence in Te/MoS_2_ heterojunctions. Log-log plots of integrated EL intensity versus drain-source current density (*J*_ds_), with red and blue solid lines indicating power-law fits to different current regimes. **a** Variation under different source-drain bias, showing distinct low- and high-current-density scaling. **b** Variation under different gate voltages, exhibiting segmented power-law behavior with fitted exponents reported in the text. **c** External quantum efficiency (EQE) versus *J*_ds_; red and blue curves correspond to the bias sweep and gate sweep configurations in (**a** and **b**), respectively

To gain further insight into the gate-dependent EL behavior, we investigated whether the underlying radiative transitions responsible for the observed EL remain constant across different gate voltages. As shown in Fig. 4d, the emission peak position remains fixed at 3.5 μm with negligible linewidth variation, indicating a stable recombination pathway governed by band-edge transitions in Te, even as the intensity varies. This confirms that gate modulation primarily affects the carrier injection efficiency rather than the fundamental nature of the recombination mechanism in Te. Together with the bias-invariant spectrum shown in Fig. 3b, these results underscore the robustness of Te band-edge transitions against electrical perturbations. In addition, the EL polarization state remains essentially unchanged during back‑gate sweeps at a fixed drain bias. As shown in Supplementary Fig. S10, the extracted degree of linear polarization (DOP) stays within ~0.58–0.59 over a broad gate range while the EL intensity varies, demonstrating a polarization‑locked intensity control enabled by electrostatic tuning. In contrast to materials such as BP, whose emission-energy shifts with applied vertical fields^33^, the gate bias in our Te/MoS_2_ heterojunction modulates EL intensity without inducing measurable changes in emission wavelength, demonstrating stable band-edge emission under field control. To further establish the gate dependence of EL, we measured the EL intensity as a function of *V*_g_, as shown in Fig. 4e. As the gate voltage is swept from positive toward negative values, the EL intensity decreases rapidly and is effectively quenched. Based on the EL-*V*_g_ intensity curve shown in Fig. 4e, the EL threshold is more precisely identified as *V*_g-th_ = −2.7 V. The refined threshold provides a clear reference point for subsequent analysis of the current-dependent EL efficiency (see Fig. 5).

### Driving-current dependence of mid-infrared electroluminescence

To probe how the bias and gate voltage affect the internal quantum efficiency and carrier injection efficiency, we extract the differential power-law exponent *k* from log-log plots of *I*_EL_ vs $J_{\mathrm{ds}}\,({\mathrm{calculated}}\, {\mathrm{using}}\, {\mathrm{a}}\, {\mathrm{junction}}\, {\mathrm{area}}\, A \sim 300\mu{\mathrm{m}}^{2},\, J_{{\mathrm{ds}}}=I_{{\mathrm{ds}}}/A)$_._ Following the k-analysis commonly used for LEDs, the integrated EL intensity can be written as the product of the source-drain current term (${I}_{{\rm{ds}}}$), the injection efficiency (${\eta }_{{\rm{inj}}}$), and the internal quantum efficiency (${\eta }_{{\rm{IQE}}}$)^51–53^, so that

1 $${I}_{{\rm{EL}}}\propto {I}_{{\rm{ds}}}\cdot {\eta }_{{\rm{inj}}}\cdot {\eta }_{{\rm{IQE}}}$$

In the active-region-limited regime, this leads to

2 $$k=\frac{{\rm{d}}{\mathrm{ln}}{{I}}_{{\rm{EL}}}}{{\rm{d}}\,{\mathrm{ln}}\,{I}_{{\rm{ds}}}}={1+\frac{{\rm{dln}}{\eta }_{{\rm{IQE}}}}{{\rm{d}}{\mathrm{ln}}\,{I}_{{\rm{ds}}}}}+\frac{{\rm{dln}}{\eta }_{{\rm{inj}}}}{{\rm{d}}{\mathrm{ln}}\,{I}_{{\rm{ds}}}}={k}_{\perp }+\frac{{\rm{dln}}{\eta }_{{\rm{inj}}}}{{\rm{d}}\,{\mathrm{ln}}\,{I}_{{\rm{ds}}}}$$

where ${\eta }_{{\rm{inj}}}$ characterizes the effective injection efficiency of carriers that can cross the Te/MoS_2_ interface barrier and enter the Te recombination zone. This term accounts for current losses before radiative recombination in Te, including nonradiative current paths in the MoS_2_ access region and interfacial nonradiative recombination. ${k}_{\perp }$ reflects the competition between radiative and nonradiative processes inside Te. In conventional LED recombination models, Shockley-Read-Hall (SRH) defect recombination, band-to-band radiative recombination, and Auger recombination correspond to characteristic values of *k*_⊥_$\approx$2, 1, and 2/3, respectively^54–56^. Experimentally, we sweep ${V}_{{\rm{ds}}}$ at a fixed gate voltage (${V}_{{\rm{g}}}=20$V, Fig. 5a) and sweep ${V}_{{\rm{g}}}$ at a fixed bias (${V}_{{\rm{ds}}}=4\mathrm{V}$, Fig. 5b) to study the dependence of EL (${I}_{{\rm{EL}}}$) on the drain current (${I}_{{\rm{ds}}})$.

Figure 5a shows ${I}_{{\rm{EL}}}$ versus current density ${J}_{{\rm{ds}}}$ on a double-logarithmic scale at ${V}_{{\rm{g}}}=20{\rm{V}}$, a bias chosen to maximize the EL signal-to-noise ratio. Between the EL onset at ${J}_{{\rm{ds}}}\approx 0.05\,{\rm{\mu }}{\rm{A}}{{\rm{\mu }}{\rm{m}}}^{-2}$ and ${J}_{{\rm{ds}}}\approx 0.4\,{\rm{\mu }}{\rm{A}}\,{{\rm{\mu }}{\rm{m}}}^{-2}$, the dependence is super-quadratic with an effective exponent $k\approx 2.36$. This large *k* indicates that defect-assisted SRH recombination in Te remains the dominant recombination channel^55–57^. As *V*_ds_ increases, the Te/MoS_2_ barrier is lowered and thinned, increasing the fraction of the total current, I_ds_, injected into Te. In the framework of Eq. (1), this corresponds to a positive $d\left(\mathrm{ln}\,{\eta }_{{\rm{inj}}}\right)/d\left(\mathrm{ln}\,{I}_{{\rm{ds}}}\right)$, which drives the measured exponent slightly above the SRH limit. For higher currents, ${0.4{\rm{\mu }}{\rm{A}}{\rm{\mu }}{\rm{m}}}^{-2}\lesssim {J}_{{\rm{ds}}}\lesssim 1.3\,{\rm{\mu }}{\rm{A}}\,{{\rm{\mu }}{\rm{m}}}^{-2}$, the slope decreases to a super-linear $k\approx 1.29$. In this regime, radiative recombination in Te becomes dominant (~1)^56,57^^,^ and the injection efficiency continues to improve with increasing *V*_ds_, so *k* remains slightly above 1.

To separate the effects of forward bias and gate control while maintaining a high signal-to-noise ratio, we next fix ${V}_{{\rm{ds}}}=4\,{\rm{V}}$ and tune ${V}_{{\rm{g}}}$ (Fig. 5b). When the gate-induced current lies in the range ${J}_{{\rm{ds}}}\approx 0.03$–$0.3\,{\rm{\mu }}{\rm{A}}\,{{\rm{\mu }}{\rm{m}}}^{-2}$, ${I}_{{\rm{EL}}}$ again follows a super-linear power law with $k\approx 1.89$. This value indicates that defect-related SRH recombination in the active region remains important, so *k*_⊥_ is close to the SRH limit (~2)^55–57^. In this regime, increasing *V*_g_ at fixed ${V}_{{\rm{ds}}}$ = 4 V rapidly increases the MoS_2_ channel conductance and raises the effective Te/MoS_2_ junction barrier. A smaller fraction of I_ds_ reaches the radiative zone, and a larger fraction is dissipated via nonradiative or interfacial dark paths outside the EL-active region. In the language of Eq. (1), ${\eta }_{{\rm{inj}}}$ decreases with ${I}_{{\rm{ds}}}$, and this negative trend lowers the measured *k* from the SRH-like value. For a higher current $0.3\,{\rm{\mu }}{\rm{A}}\,{{\rm{\mu }}{\rm{m}}}^{-2}\lesssim {J}_{{\rm{ds}}}\lesssim 1.3\,{{\mu }}{\rm{A}}\,{{{\mu }}{\rm{m}}}^{-2}$, the slope drops to $k\approx 0.71$. In this regime, radiative recombination in Te remains relevant^56,57^, but the gate-induced redistribution of potential promotes interfacial nonradiative recombination and other loss pathways outside the EL-active zone. As a result, *η*_inj_ decreases with increasing *I*_ds_, providing a negative contribution to ${\rm{d}}\left(\mathrm{ln}\,{\eta }_{{\rm{inj}}}\right)/{\rm{d}}\left(\mathrm{ln}\,{I}_{{\rm{ds}}}\right)$ and causing the measured *k* to fall below 1. At the largest drive currents (${J}_{{\rm{ds}}}\gtrsim 1.3\,{\rm{\mu }}{\rm{A}}\,{{\rm{\mu }}{\rm{m}}}^{-2}$), the EL intensity eventually rolls over and decreases with increasing ${J}_{{\rm{ds}}}$, corresponding to an effective negative exponent in the ${I}_{{\rm{EL}}}$-${J}_{{\rm{ds}}}$ power-law relation.

These trends can be consistently interpreted within a standard LED framework that separates injection efficiency from the internal quantum efficiency of the Te active region. In the low-current-density window ($0.03$–$0.3\,{\rm{\mu }}{\rm{A}}\,{{\rm{\mu }}{\rm{m}}}^{-2}$), back-gating accumulates electrons in the MoS_2_ edge/access regions and lowers the effective injection barrier into Te, thereby suppressing the SRH-type losses in Te and increasing ${\eta }_{{\rm{inj}}}$ with current; together, these effects naturally yield super-linear exponents approaching the SRH-like limit. As ${V}_{{\rm{g}}}$ increases further, the junction enters a regime in which gate-induced band bending on the MoS_2_ side becomes substantial (cf. Fig. 2), a larger fraction of ${I}_{{\rm{ds}}}$ is either diverted into interfacial nonradiative recombination and other leakage pathways outside the Te radiative zone or shunted through the MoS_2_ rather than recombining in Te. In other words, ${\eta }_{{\rm{inj}}}$ begins to decrease with current $({\rm{dln}}{\eta }_{{\rm{inj}}}/{\rm{dln}}{I}_{{\rm{ds}}} < 0)$, which by itself can drive the observed sub-linear behavior ($k\approx 0.71$) even if radiative recombination remains important in Te. At the highest drive currents (${J}_{{\rm{ds}}}\gtrsim 1.3\,{\rm{\mu }}{\rm{A}}\,{{\rm{\mu }}{\rm{m}}}^{-2}$), the slope *k* dips and becomes negative. As the carrier density increases, high-order nonradiative processes such as Auger recombination become significant, and self-heating further enhances nonradiative loss. The gate-induced barrier, together with interfacial dark recombination and other leakage pathways, continues to reduce the fraction of *I*_ds_ that reaches the EL-active region, so *k*_⊥_ tends toward the Auger value (~2/3)^55,58,59^, while ${\rm{dln}}{\eta }_{{\rm{inj}}}/{\rm{dln}}{I}_{{\rm{ds}}}$ remains negative; together, these factors drive the overall *k* below zero.

To quantify the device efficiency, we evaluate the external quantum efficiency (EQE) of the Te/MoS_2_ heterojunction LED. EQE is defined as the ratio of photons emitted into the far field to electrons passing through the device, i.e., ${\rm{EQE}}={\varPhi }_{\mathrm{ph}}/\left({I}_{\mathrm{ds}}/{\rm{q}}\right)$. The photon flux ${\varPhi }_{\mathrm{ph}}$ is obtained from the measured EL intensity after correcting for the calibrated throughput of the collection optics and detector, as detailed in Supplementary Note 5. Figure 5c shows the EQE as a function of drive current ${I}_{\mathrm{ds}}$ for the same device, where the red curve corresponds to sweeping ${V}_{\mathrm{ds}}$ at fixed ${V}_{{\rm{g}}}=20\,\mathrm{V}$ and the blue curve corresponds to sweeping ${V}_{{\rm{g}}}$ at fixed ${V}_{\mathrm{ds}}=4\,\mathrm{V}$. Within our IQE-injection-efficiency framework, the integrated EL intensity scales as ${I}_{\mathrm{EL}}\propto {I}_{\mathrm{ds}}{\eta }_{\mathrm{inj}}\,{\eta }_{\mathrm{IQE}}$, which yields:

3 $$d\,{\mathrm{ln}}\left({\mathrm{EQE}}\right)/{\rm{d}}\,{\mathrm{ln}}\left({I}_{\mathrm{ds}}\right)=k-1$$

Thus, *k* > 1 indicates that the EQE increases with current, *k* ≈ 1 marks the vicinity of the EQE maximum, and *k* < 1 signals the onset of efficiency droop. For the forward-bias sweep at fixed ${V}_{{\rm{g}}}=20\,\mathrm{V}$ (red curve), the EQE continues to increase with *J*_ds_ over the measured range, as expected from the super-linear exponents *k* > 1 (Fig. 5a). Under these conditions, a maximum EQE of ~0.10% is obtained. In contrast, for the gate sweep at fixed ${V}_{\mathrm{ds}}=4\,\mathrm{V}$ (blue curve), the EQE initially rises steeply with increasing *J*_ds_, reaches a peak (EQE ≈ 0.32%) near the current where *k* ≈ 1, and then decreases to ~0.10% as *k* drops below unity and eventually becomes negative (Fig. 5b). The shapes and peak positions of the EQE-*J*_ds_ trace closely mirror the segmented *k* value behavior discussed above, providing a consistency check of the combined IQE-injection-efficiency picture. In this three-terminal heterojunction, the drain bias and the gate voltage act as two independent knobs for controlling the EL output. The drain bias mainly sets the overall forward-bias drive and thus the injection level, which naturally modulates the EL intensity. In contrast, the back gate electrostatically tunes the MoS_2_ carrier density, altering the resistance and, consequently, the injection current. However, the distinct EQE-*J*_ds_ curves for *V*_ds_-sweep and *V*_g_-sweep in Fig. 5c indicate that the back gate provides additional control over the device: it can redistribute the potential drop near the Te/MoS_2_ junction, thereby modifying the effective interfacial injection condition and the radiative probability per injected carrier. As a result, gating can shift the device into a higher-efficiency operating window and enhance the EQE without requiring a proportional increase in *J*_ds_. Notably, a peak EQE of ~0.32% is achieved in a simple planar device geometry without any optical cavity, reflective back contact, or waveguide-based light extraction. In light of recent reports on mid-infrared BP-based LEDs employing resonant cavities and reflective electrodes^28,60^, we anticipate that similar optical and interfacial engineering could further enhance the EQE of Te/MoS_2_ heterojunction LEDs.

## Discussion

In summary, we demonstrate a gate-tunable Te/MoS_2_ van der Waals LED emitting at ~3.5 μm, a technologically important mid-IR wavelength, with linearly polarized output and spectra that remain invariant to bias and gate at 25 K. The device architecture enables electrostatic control of band alignment and injection efficiency, while a segmented k-slope analysis identifies injection-efficiency droop as the principal limitation at high drive. These results position the Te/MoS_2_ heterostructure as a polarization-sensitive MIR source compatible with heterogeneous integration, with immediate implications for on-chip sensing, spectroscopy, and communications in the 3–5 μm window. In particular, polarization-sensitive MIR emission and the potential for integration with mature photonics platforms open opportunities for compact, chip-scale spectrometers, trace-gas sensing, environmental monitoring, and polarized MIR communication systems that leverage atmospheric transmission windows.

Looking ahead, optimizing growth methods to produce high-quality Te crystals with lower defect densities and cleaner interfaces will minimize nonradiative recombination and improve carrier-injection efficiency, thereby significantly enhancing quantum efficiency. Building on these material advances, higher brightness and more stable operation could be pursued through contact and barrier engineering, thermal management, and pulsed operation to enable room-temperature performance. Photonic confinement strategies, such as microcavities, waveguide-coupled resonators, and photonic crystals, could further enhance out-coupling. Such photonic designs could improve on-chip compatibility by redirecting the emission of the present simple LED geometry into guided or waveguide-coupled modes^27,61^. Realization on CMOS-friendly substrates and co-integration with Si or other MIR photonics platforms would enable lab-on-a-chip sensing, portable MIR spectrometers, and field-deployable LIDAR or free-space links^62–64^. Beyond this system, the segmented k-slope framework provides a generalizable toolkit for vdW emitters and can guide the rational design of reconfigurable, on-chip MIR optoelectronics, including multi-color or tunable polarization-enabled sources^29,65^.

## Materials and methods

### Sample preparation

Te nanoflakes were grown through a hydrothermal method^38,39^. Initially, 3 g of polyvinylpyrrolidone (PVP, molecular weight = 58,000) was dissolved in 32 mL of deionized (DI) water. Subsequently, 92 mg of Na_2_TeO_3_ was put into the PVP solution with continuous stirring. Next, 3.32 mL of ammonium hydroxide solution (25–28%, wt/wt%) and 1.68 mL of hydrazine hydrate (80%, wt/wt%) were added to the mixture. After five minutes of magnetic stirring, the solution was transferred into a 50 mL Teflon-lined stainless-steel autoclave and maintained at 180 °C for 10 h. The resulting product was washed thoroughly with deionized water to remove any residual ions.

For device fabrication, the Te nanoflakes were redispersed in ethyl alcohol and drop-cast onto a 285-nm SiO_2_/Si substrate. Multilayer MoS_2_ flakes were exfoliated using polydimethylsiloxane (PDMS) and transferred onto selected Te flakes via a dry transfer method. Electrode fabrication was carried out in two sequential electron beam lithography (EBL) steps. First, electrodes were patterned on the Te flake, followed by electron beam evaporation of 20 nm Pd and 100 nm Au (Pd/Au). In the second EBL step, electrodes were defined on the MoS_2_ region, and a 20-nm Cr and 100-nm Au (Cr/Au) metal stack was deposited using the same electron beam evaporation process. Each lithography and deposition step was followed by a standard lift-off process in acetone.

### Mid-infrared EL measurement

EL measurements were performed using a self-built micro-EL setup. The sample was mounted in an open‑loop cryogenic thermostat providing a vacuum environment and temperature control from 25 to 300 K via a liquid helium flow. The cryostat was fixed onto a PDV XY‑50 motorized XY scanning stage to enable spatially resolved measurements. A × 40 reflective objective lens with a numerical aperture of 0.5 was used to collect the EL emission, which was directed into a spectrometer (Princeton Instruments SP2500i) equipped with a 150 g mm^−1^ grating. The signal was detected using a liquid-nitrogen-cooled single-channel InSb photodetector. To improve the signal-to-noise ratio, the EL signal was modulated by an optical chopper at 521 Hz and demodulated using a lock-in amplifier phase-locked to the chopper reference. The optical chopper was placed in the collection path. During the measurements, the electrical bias was applied using a Keithley 2612B dual-channel source meter, with one channel supplying the back-gate voltage and the other applying the drain-source bias.

### PL and Raman measurement

For photoluminescence (PL) measurements, the same low-temperature cryogenic setup, motorized XY stage, and lock-in detection scheme described for EL were used. A 1064 nm continuous-wave laser was employed as the excitation source. The PL signal was collected by the same objective and detected using the same InSb photodetector. For Raman spectroscopy, a 532-nm laser was used for excitation, and the scattered signal was collected by the same spectrometer with the same grating and detected using a CCD camera.

## Supplementary information

Supplementary Information

## Data Availability

The data that support the findings of this study are available from the corresponding author upon reasonable request.

## References

[1] De Palo, R. et al. Femtosecond laser fabrication of black quartz for infrared photodetection applications. *Light Adv. Manuf.* **6**, 26 (2025).

[2] Xiong, H. et al. Ultraviolet-C to mid-infrared supercontinuum generation in periodically poled lithium tantalate waveguides. *Light Sci. Appl.* **15**, 253 (2026). 42191700 10.1038/s41377-026-02323-4 PMC13212705

[3] Wang, M. et al. Spin-orbit-locked hyperbolic polariton vortices carrying reconfigurable topological charges. *eLight* **2**, 12 (2022).

[4] Nash, G. R. et al. InSb∕AlInSb quantum-well light-emitting diodes. *Appl. Phys. Lett.* **88**, 051107 (2006).

[5] Krier, A. et al. Room temperature midinfrared electroluminescence from GaInAsSbP light emitting diodes. *Appl. Phys. Lett.* **90**, 211115 (2007).

[6] Wang, Y. P. et al. 4.7 μm mid-wave infrared quantum cascade laser with double active region structure. *Chin. Opt.* **17**, 1042–1049 (2024).

[7] Faist, J. et al. Quantum cascade laser. *Science* **264**, 553–556 (1994). 17732739 10.1126/science.264.5158.553

[8] Koerperick, E. J. et al. InAs∕GaSb cascaded active region superlattice light emitting diodes for operation at 3.8 μm. *Appl. Phys. Lett.* **92**, 121106 (2008).

[9] Provence, S. R. et al. High power cascaded mid-infrared InAs/GaSb light emitting diodes on mismatched GaAs. *J. Appl. Phys.* **118**, 123108 (2015).

[10] Schäfer, N. et al. High efficiency mid-infrared interband cascade LEDs grown on low absorbing substrates emitting >5 mW of output power. *Opt. Eng.* **58**, 117106 (2019).

[11] Shen, X. Y., Kamath, A. & Guyot-Sionnest, P. Mid-infrared cascade intraband electroluminescence with HgSe–CdSe core–shell colloidal quantum dots. *Nat. Photonics* **17**, 1042–1046 (2023).

[12] Shen, X. Y., Peterson, J. C. & Guyot-Sionnest, P. Mid-infrared HgTe colloidal quantum dot LEDs. *ACS Nano* **16**, 7301–7308 (2022). 35349280 10.1021/acsnano.2c01694

[13] Lien, D. H. et al. Large-area and bright pulsed electroluminescence in monolayer semiconductors. *Nat. Commun.* **9**, 1229 (2018). 29581419 10.1038/s41467-018-03218-8 PMC5955902

[14] Sundaram, R. S. et al. Electroluminescence in single layer MoS_2_. *Nano Lett.* **13**, 1416–1421 (2013). 23514373 10.1021/nl400516a

[15] Cheng, R. et al. Electroluminescence and photocurrent generation from atomically sharp WSe_2_/MoS_2_ heterojunction *p–n* diodes. *Nano Lett.* **14**, 5590–5597 (2014). 25157588 10.1021/nl502075n PMC4189621

[16] Murali, K. et al. Accurate extraction of Schottky barrier height and universality of Fermi level de-pinning of van der Waals contacts. *Adv. Funct. Mater.* **31**, 2010513 (2021).

[17] Li, D. H. et al. Electric-field-induced strong enhancement of electroluminescence in multilayer molybdenum disulfide. *Nat. Commun.* **6**, 7509 (2015). 26130491 10.1038/ncomms8509 PMC4507000

[18] Withers, F. et al. Light-emitting diodes by band-structure engineering in van der Waals heterostructures. *Nat. Mater.* **14**, 301–306 (2015). 25643033 10.1038/nmat4205

[19] Ross, J. S. et al. Electrically tunable excitonic light-emitting diodes based on monolayer WSe_2_ p–n junctions. *Nat. Nanotechnol.* **9**, 268–272 (2014). 24608230 10.1038/nnano.2014.26

[20] Fang, X. M. et al. Bi_2_O_3_/Bi_2_S_3_ heterojunction composite preparation and photodetection performance. *Chin. Opt.* **18**, 1036–1043 (2025).

[21] Shi, X. Q. et al. Room-temperature polarized light-emitting diode-based on a 2D monolayer semiconductor. *Small* **19**, 2301949 (2023). 10.1002/smll.202301949 37357166

[22] Yuan, H. T. et al. Polarization-sensitive broadband photodetector using a black phosphorus vertical p–n junction. *Nat. Nanotechnol.* **10**, 707–713 (2015). 26030655 10.1038/nnano.2015.112

[23] Li, L. K. et al. Direct observation of the layer-dependent electronic structure in phosphorene. *Nat. Nanotechnol.* **12**, 21–25 (2017). 27643457 10.1038/nnano.2016.171

[24] Zhang, G. W. et al. Infrared fingerprints of few-layer black phosphorus. *Nat. Commun.* **8**, 14071 (2017). 28059084 10.1038/ncomms14071 PMC5227111

[25] Huang, L. et al. Waveguide-integrated black phosphorus photodetector for mid-infrared applications. *ACS Nano* **13**, 913–921 (2019). 30586289 10.1021/acsnano.8b08758

[26] Wang, J. J. et al. Mid-infrared polarized emission from black phosphorus light-emitting diodes. *Nano Lett.* **20**, 3651–3655 (2020). 32286837 10.1021/acs.nanolett.0c00581

[27] Chang, T. Y. et al. Black phosphorus mid-infrared light-emitting diodes integrated with silicon photonic waveguides. *Nano Lett.* **20**, 6824–6830 (2020). 32816495 10.1021/acs.nanolett.0c02818

[28] Gupta, N. et al. Bright mid-wave infrared resonant-cavity light-emitting diodes based on black phosphorus. *Nano Lett.* **22**, 1294–1301 (2022). 35072481 10.1021/acs.nanolett.1c04557

[29] Chen, P. L. et al. Van der Waals heterostructure mid-infrared emitters with electrically controllable polarization states and spectral characteristics. *ACS Nano* **17**, 10181–10190 (2023). 37212535 10.1021/acsnano.3c00277

[30] Gupta, N. et al. Large-scale efficient mid-wave infrared optoelectronics based on black phosphorus ink. *Sci. Adv.* **9**, eadi9384 (2023). 38064551 10.1126/sciadv.adi9384 PMC10708190

[31] Wood, J. D. et al. Effective passivation of exfoliated black phosphorus transistors against ambient degradation. *Nano Lett.* **14**, 6964–6970 (2014). 25380142 10.1021/nl5032293

[32] Zong, X. R. et al. Black phosphorus-based van der Waals heterostructures for mid-infrared light-emission applications. *Light Sci. Appl.* **9**, 114 (2020). 32637081 10.1038/s41377-020-00356-x PMC7329856

[33] Kim, H. et al. Actively variable-spectrum optoelectronics with black phosphorus. *Nature* **596**, 232–237 (2021). 34381234 10.1038/s41586-021-03701-1

[34] Qiu, G. et al. The resurrection of tellurium as an elemental two-dimensional semiconductor. *npj 2D Mater. Appl.* **6**, 17 (2022).

[35] Pan, Y. Y. et al. Dependence of excited-state properties of tellurium on dimensionality: from bulk to two dimensions to one dimensions. *Phys. Rev. B* **98**, 085135 (2018).

[36] Shi, Z. et al. Two-dimensional tellurium: progress, challenges, and prospects. *Nano Micro Lett.* **12**, 99 (2020). 10.1007/s40820-020-00427-z PMC7770852 34138088

[37] Li, P. K. Mid-infrared photoconductivity spectra of single tellurium nanowires. *J. Appl. Phys.* **128**, 063105 (2020).

[38] Amani, M. et al. Solution-synthesized high-mobility tellurium nanoflakes for short-wave infrared photodetectors. *ACS Nano* **12**, 7253–7263 (2018). 29912549 10.1021/acsnano.8b03424

[39] Wang, Y. X. et al. Field-effect transistors made from solution-grown two-dimensional tellurene. *Nat. Electron.* **1**, 228–236 (2018).

[40] Tong, L. et al. Stable mid-infrared polarization imaging based on quasi-2D tellurium at room temperature. *Nat. Commun.* **11**, 2308 (2020). 32385242 10.1038/s41467-020-16125-8 PMC7210936

[41] Li, J. J. et al. Polarity-reversible Te/WSe_2_ van der Waals heterodiode for a logic rectifier and polarized short-wave infrared photodetector. *ACS Appl. Mater. Interfaces* **14**, 53202–53212 (2022). 36395442 10.1021/acsami.2c17331

[42] Cheng, Y. et al. Multifunctional optoelectronic devices based on two-dimensional tellurium/MoS_2_ heterojunction. *Appl. Phys. Lett.* **125**, 171105 (2024).

[43] Wang, S. et al. Mid-infrared photoluminescence from tellurium thin films. *Nano Lett.* **25**, 9311–9317 (2025). 40454649 10.1021/acs.nanolett.5c01622

[44] Zhang, J. R. et al. Dynamically tunable polarized mid-infrared light-emitting diodes from polarization singularities in a band-edge Weyl node. *Nat. Commun.* **16**, 11213 (2025). 41387404 10.1038/s41467-025-66014-1 PMC12715210

[45] Ma, J. C. et al. Unveiling Weyl-related optical responses in semiconducting tellurium by mid-infrared circular photogalvanic effect. *Nat. Commun.* **13**, 5425 (2022). 36109522 10.1038/s41467-022-33190-3 PMC9477843

[46] Liang, D. L. et al. Topologically enhanced giant broadband second-harmonic generation in Weyl semiconductor tellurium. *Nat. Commun.* **16**, 10393 (2025). 41285863 10.1038/s41467-025-65353-3 PMC12644508

[47] Liang, D. L. et al. Electrically tunable and linearly polarized mid-infrared photoluminescence in 2D tellurium. *Adv. Mater.* **38**, e17175 (2026). 41452193 10.1002/adma.202517175

[48] Cheng, B. et al. Topological field-effect transistor based on quasi-two-dimensional tellurium flakes. *Phys. Rev. Appl.* **17**, 054044 (2022).

[49] Lee, S. Y. et al. Large work function modulation of monolayer MoS_2_ by ambient gases. *ACS Nano* **10**, 6100–6107 (2016). 27232340 10.1021/acsnano.6b01742

[50] Nouchi, R. Edge-induced Schottky barrier modulation at metal contacts to exfoliated molybdenum disulfide flakes. *J. Appl. Phys.* **120**, 064503 (2016).

[51] Wang, L. et al. A review on experimental measurements for understanding efficiency droop in InGaN-based light-emitting diodes. *Mater.* **10**, 1233 (2017). 10.3390/ma10111233 PMC5706180 29072611

[52] Han, D. P. et al. Analysis of nonradiative recombination mechanisms and their impacts on the device performance of InGaN/GaN light-emitting diodes. *Jpn. J. Appl. Phys.* **54**, 02BA01 (2015).

[53] Rashidi, A. et al. Thermal and efficiency droop in InGaN/GaN light-emitting diodes: decoupling multiphysics effects using temperature-dependent RF measurements. *Sci. Rep.* **9**, 19921 (2019). 31882667 10.1038/s41598-019-56390-2 PMC6934866

[54] Shim, J. I. & Shin, D. S. Measuring the internal quantum efficiency of light-emitting diodes: towards accurate and reliable room-temperature characterization. *Nanophotonics* **7**, 1601–1615 (2017).

[55] Kim, K. S. et al. Analysis of dominant carrier recombination mechanisms depending on injection current in InGaN green light emitting diodes. *Appl. Phys. Lett.* **104**, 091110 (2014).

[56] Yoo, Y. S. et al. Effective suppression of efficiency droop in GaN-based light-emitting diodes: role of significant reduction of carrier density and built-in field. *Sci. Rep.* **6**, 34586 (2016). 27756916 10.1038/srep34586 PMC5069459

[57] Baek, W. J. et al. Ultra-low-current driven InGaN blue micro light-emitting diodes for electrically efficient and self-heating relaxed microdisplay. *Nat. Commun.* **14**, 1386 (2023). 36932091 10.1038/s41467-023-36773-w PMC10023660

[58] Seetoh, I. P. et al. Auger recombination as the dominant recombination process in indium nitride at low temperatures during steady-state photoluminescence. *Appl. Phys. Lett.* **102**, 101112 (2013).

[59] Grant, P. C. et al. Auger-limited minority carrier lifetime in GeSn/SiGeSn quantum well. *Appl. Phys. Lett.* **124**, 111110 (2024).

[60] Brodeur, J. et al. Current crowding in a high-efficiency black phosphorus light-emitting diode using a reflective back contact. *Nano Lett.* **25**, 11536–11542 (2025). 40679117 10.1021/acs.nanolett.5c01829

[61] Liu, C. H. et al. Nanocavity integrated van der Waals heterostructure light-emitting tunneling diode. *Nano Lett.* **17**, 200–205 (2017). 27936763 10.1021/acs.nanolett.6b03801

[62] Meng, Y. et al. Photonic van der Waals integration from 2D materials to 3D nanomembranes. *Nat. Rev. Mater.* **8**, 498–517 (2023).

[63] Yang, D. H. et al. Dimensionality-enhanced mid-infrared light vortex detection based on multilayer graphene. *Light Sci. Appl.* **14**, 116 (2025). 40044647 10.1038/s41377-024-01735-4 PMC11882842

[64] Cheng, J. L. et al. Direct photocurrent detection of optical vortex based on the orbital photo galvanic effect: progress, challenge, and perspective. *Adv. Sci.* **13**, e19333 (2026). 10.1002/advs.202519333 PMC13170267 41944391

[65] Chen, C. et al. Widely tunable mid-infrared light emission in thin-film black phosphorus. *Sci. Adv.* **6**, eaay6134 (2020). 32110733 10.1126/sciadv.aay6134 PMC7021507

