# Supplementary material for: Gate-tunable mid-infrared electroluminescence from Te/MoS2 p-n heterojunctions
Source: Light Sci Appl. 2026 Jul 27;15:333. doi: 10.1038/s41377-026-02402-6 (PMC13402784; doi:10.1038/s41377-026-02402-6)
Supplement: Supplementary file 1 — Supplementary Information [file 41377_2026_2402_MOESM1_ESM.docx]

Supplementary Information for

**Gate-Tunable** **Mid-Infrared Electroluminescence from Te/MoS_2_ p-n Heterojunctions**

Shiyu Wang (王时雨)^1^, Delang Liang (梁德琅)^1,2^, Zhi Zheng (郑直)^3^, Mingyang Qin (秦明洋)^1^, Yuchun Chen (陈煜淳)^1^, Jie Sheng (盛杰)^1^, Shula Chen (陈舒拉)^2^, Lin Li (李林)^3^, Changgan Zeng (曾长淦)^3^, Anlian Pan (潘安练)^2,4†^, Jinluo Cheng (程晋罗)^5,6†^, and Dong Sun (孙栋)^1,6,7,8†^

^1^International Center for Quantum Materials, School of Physics, Peking University, Beijing, China.

^2^Key Laboratory for Micro-Nano Physics and Technology of Hunan Province, Hunan Institute of Optoelectronic Integration, College of Materials Science and Engineering, Hunan University, Changsha, China.

^3^CAS Key Laboratory of Strongly Coupled Quantum Matter Physics, and Department of Physics, University of Science and Technology of China, Hefei, China.

^4^School of Physics and Electronics, Hunan Normal University, Changsha, China.

^5^GPL Photonics Laboratory, State Key Laboratory of Luminescence Science and Applications, Changchun Institute of Optics Fine Mechanics and Physics, Chinese Academy of Sciences, Changchun, China.

^6^School of Physics and Laboratory of Zhongyuan Light, Zhengzhou University, Zhengzhou, China.

^7^Collaborative Innovation Center of Quantum Matter, Beijing, China.

^8^ Frontiers Science Center for Nano-optoelectronics, School of Physics, Peking University, Beijing, China.

^†^Email: sundong@pku.edu.cn; jlcheng@ciomp.ac.cn; anlian.pan@hnu.edu.cn;

List of contents:

Supplementary figures:

Fig. S1. Thickness of the MoS_2_/Te device. 3

Fig. S2. Electrical characterization at 25 K. 4

Fig. S3. Power-dependent PL and log-log analysis. 5

Fig. S4. Gate-voltage dependence of EL at 25 K, 40 K, and 80 K. 6

Fig. S5. Spatial mapping of mid-infrared EL and 1064-nm reflection maps. 8

Fig. S6. Comparison of polarization characteristics for two Te/TMD heterojunction emitters measured at 25 K. 10

Fig. S7. Polarization-resolved PL of isolated Te flakes at 25 K 11

Fig. S8. Long-term stability. 12

Fig. S9. Gate‑voltage dependence of integrated EL intensity for multiple Te/TMD heterojunction LEDs measured at 25 K. 13

Fig. S10. Gate‑voltage dependence of the degree of linear polarization (DOP) in a Te/MoS_2_ heterojunction LED measured at 25 K with fixed drain bias (*V*_ds_ ~3.5 V). 14

Fig. S11. Extracted EL full width at half maximum under bias and gate modulation 15

**Supplementary Note 1. Optical image and AFM thickness mapping**

Fig. S1a identifies the device layout and the AFM scan window (yellow dashed box); the inset shows the AFM scan from which two representative line profiles were taken along the cyan and green dashed lines. Fig. S1b plots the corresponding AFM height traces, with the upper (cyan) profile crossing the MoS_2_ terrace edge and the lower (purple) profile crossing the Te step, yielding thicknesses of 17 nm for MoS_2_ and 234 nm for Te, as quoted in the main text. The AFM provides a geometric reference for the optical and electrical measurements reported in the manuscript. The Te flake used in this device is 234 nm thick. We selected Te flakes by screening the PL response of multiple candidates. By PL characterization of multiple Te flakes from the same hydrothermal batch, we found that the thinnest flakes often show less robust morphology and lower emission yield. Therefore, we chose Te flakes with sufficiently uniform morphology to ensure stable junction formation and reliable EL operation. For the n-type injecting layer, we use relatively thick MoS_2_ (~17 nm) rather than a monolayer or thin layer to ensure robust electronic transport and adequate current drive under practical contact configurations. This choice helps reduce the effective series resistance and enables reproducible EL turn-on. On the other hand, electrostatic screening generally becomes stronger as the thickness increases; therefore, a relatively thin MoS_2_ layer is required to preserve effective gate tunability of the device.

**
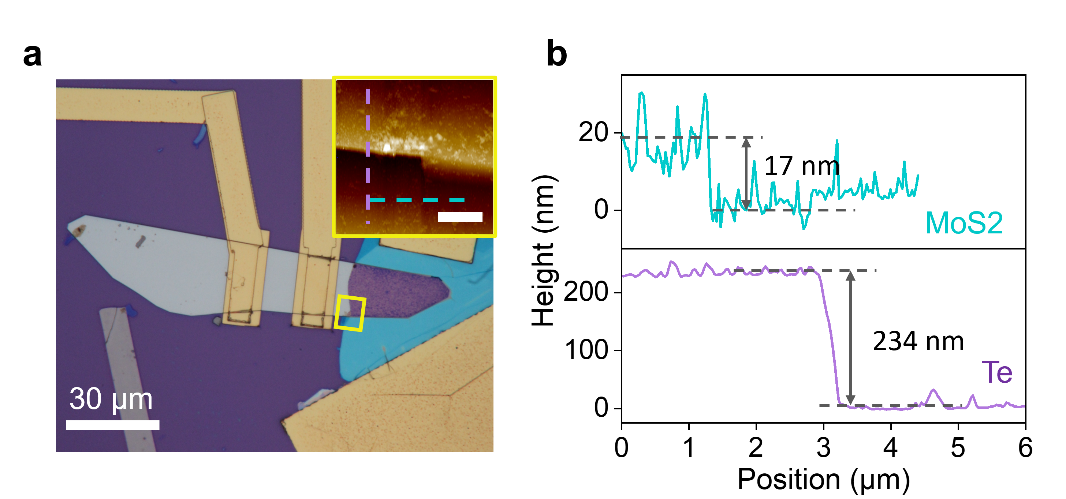
**

Fig. S1. Thickness of the MoS_2_/Te device. **a,** Optical image of the heterostructure; the yellow rectangle marks the region imaged by AFM (inset). In the AFM inset, cyan and purple dashed guide lines indicate the locations of the line traces plotted in b. **b,** AFM height profiles (height vs position) scanning along the dashed lines marked in panel a (inset): the upper cyan trace shows the MoS_2_ thickness, and the lower purple trace shows the Te thickness. The step heights are consistent with the values given in the main text. Scale bars: 30 μm (a), 1 μm (AFM inset).

**Supplementary Note 2. Low-temperature transport of Te and Te/MoS₂**

Fig. S2 presents the 25 K transport measurements; the Te output characteristics, Te transfer characteristics, and Te/MoS_2_ heterojunction transfer characteristics are presented in panels a–c, respectively. For the isolated Te flake (panels a–b), a nearly linear small-bias $I_{\text{ds}}-V_{\text{ds}}$ response was observed (Ohmic contacts), and an effective small-bias resistance of $\sim2.3 \text{kΩ}$ was extracted; the $I_{\text{ds}}-V_{g}$ curve decreased monotonically with $V_{g}$ consistent with p-type conduction. Its transfer curve exhibited weak back-gate modulation at 25 K, changing from $\sim6 \mu\text{A}$ at $V_{g}=-60 \text{V}$ to $3.7 \mu\text{A}$ at $V_{g}=+60 \text{V}$, consistent with strong electrostatic screening in the Te flake, and consequently, the gate modulation was very limited.

For the Te/MoS_2_ heterojunction (panel c), transfer curves acquired at $V_{\text{ds}}=+4,+2, \text{and}-4 \text{V}$ revealed gate-dependent turn-on in forward bias, whereas the reverse-bias branch remains suppressed. A small reverse current was also observed at $V_{\text{ds}}=-4 \text{V}$ near $V_{g}=+30 \text{V}$. This asymmetry is attributed to back-gating that primarily tunes the MoS_2_ Fermi level (thin and strongly coupled), whereas the much thicker Te is electrostatically screened; as a result, the interfacial barrier and forward injection are modulated, while the basic rectification remains unchanged. Because a single heterojunction transfer trace at $V_{\text{ds}}=+4 \text{V}$ is already presented in the main text, additional transfer curves ${(V}_{\text{ds}}=+2 \text{V,}-4 \text{V}\text{)}$ are included here to complete the bias-gate matrix while avoiding redundancy in the main figures.


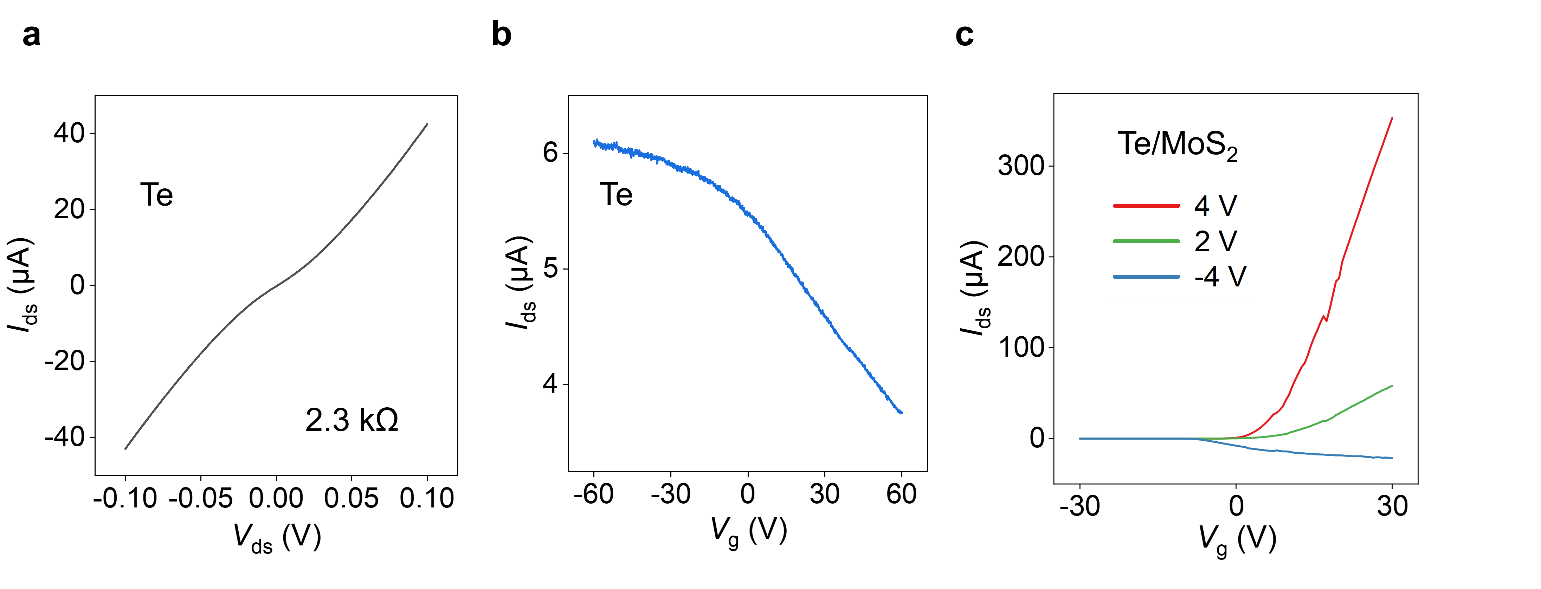


Fig. S2. Electrical characterization at 25 K. **a,** Te output characteristics, *I*_ds_ *-V*_ds_. **b,** Te transfer characteristics, *I*_ds_ -*V*_g_. **c,** Te/MoS_2_ heterojunction transfer characteristics at *V*_ds_ = +4, +2, and −4 V.

**Supplementary Note 3. Power-dependent photoluminescence**

Fig. S3a shows PL spectra from the junction region at 25 K while sweeping the excitation power from 0.53 mW to 49.5 mW. The peak position remains essentially unchanged. The integrated PL versus power (Fig. S3b) follows segmented power laws $I_{\text{PL}}\propto P^{k}$ with exponents $k_{1}=1.9$ (low power, super-linear), and $k_{2}=1.1$ (high power). The emission arises from Te in the junction region, while MoS_2_ does not contribute to the observed emission in the energy regions in these measurements. The super-linear behavior (*k* ~1.9) in the low-power region originates from trap filling (SRH) in Te. With increasing power, laser-induced heating and Auger losses reduce the incremental PL gain, so the slope k drops (*k* ~1.1). Integrated PL was obtained using the same spectral window for all power levels, and k was determined from linear fits to log-log $I_{\text{PL}}$-Power plots.


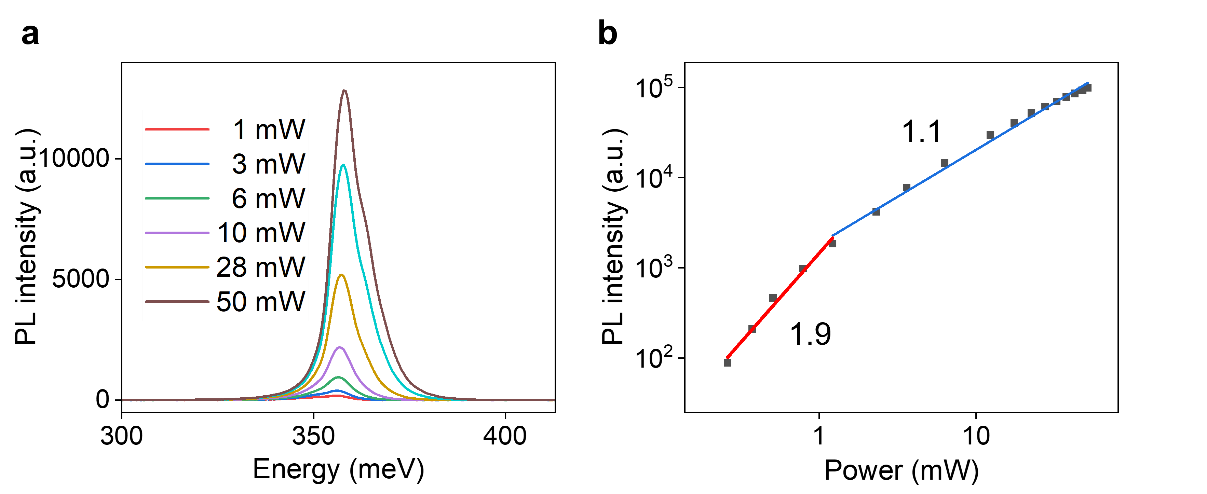


Fig. S3. Power-dependent PL and log-log analysis. **a,** PL spectra at 25 K from 0.53 mW to 49.5 mW excitation. **b,** Log-log plot of integrated PL intensity versus power with piecewise linear fits.

**Supplementary Note 4. Temperature dependence of gate-modulated electroluminescence**

Fig. S4a compares the EL integrated intensity versus $V_{g}$ at 25 K, 40 K, and 80 K for the same device and optical path. These data demonstrate that the device continues to exhibit electroluminescence up to 80 K, with the absolute intensity decreasing as temperature increases. At each temperature, the EL always rises and then falls when $V_{g}$ increases; the absolute intensity follows 25 K > 40 K > 80 K. The common trend is attributed to gate-tunable band alignment at the Te/MoS_2_ interface: back-gate modulation of the MoS_2_ Fermi level adjusts the interfacial injection barrier, while higher temperature enhances nonradiative loss and lowers the absolute intensity. Although the MoS_2_ band gap shifts with temperature (Varshni-type behavior), the overall EL-$V_{g}$ trend remains unchanged across 25–80 K, as shown in Fig. S4b. This interpretation is consistent with the non-monotonic EL -$V_{g}$ response in the main text, which shows back-gate control of the Te/MoS_2_ band alignment.


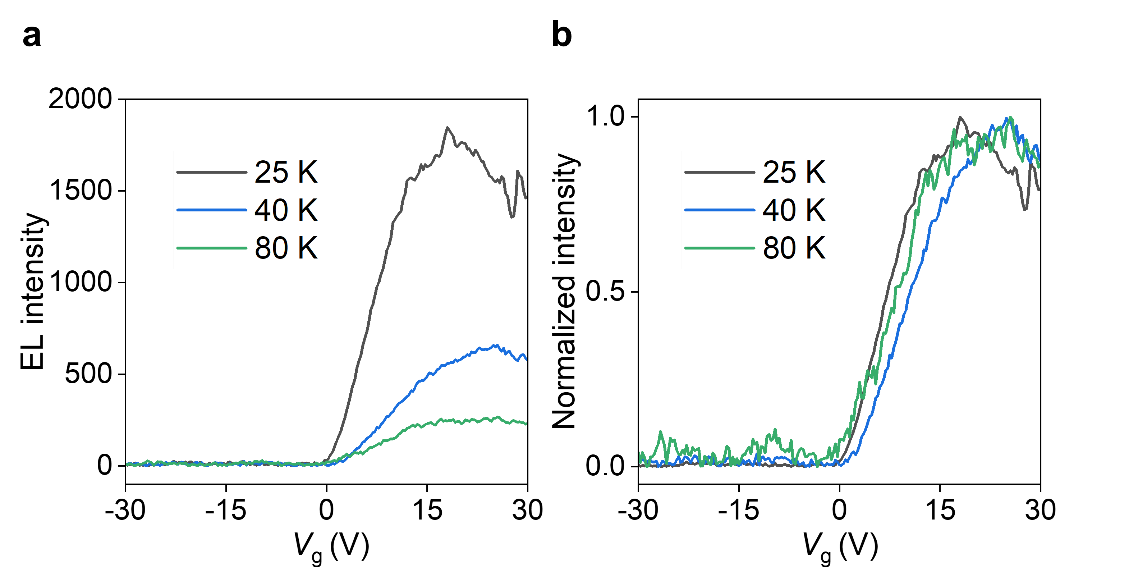


Fig. S4. Gate-voltage dependence of EL at 25 K, 40 K, and 80 K. **a,** EL integrated intensity versus *V*_g_ at 25 K, 40 K, and 80 K (same device/optical path). **b,** Normalized EL-*V*_g_ curves (25 K, 40 K, 80 K).

**Supplementary Note 5. External quantum efficiency (EQE) calibration and calculation**

In the main text, the external quantum efficiency of the Te/MoS_2_ p-n heterojunction LED is defined as the ratio between the number of photons emitted into the far field and the number of electrons passing through the device:

$$\eta_{\mathrm{EQE}}=\Phi_{\mathrm{ph}}/\left( I_{\mathrm{ds}}/q \right),$$

where $\Phi_{ph}$ is the photon flux emitted into the collection solid angle, $I_{\mathrm{ds}}$ is the measured source-drain current, and q is the elementary charge. The main task is therefore to convert the measured EL signal into an absolute photon flux.

To quantify $\Phi_{ph}$, we use a calibrated mid-infrared optical setup. The EL emission from the Te/MoS_2_ junction is collected by a reflective objective, passes through a set of mirrors, beam splitters, and relay optics, is dispersed by a monochromator, and is finally detected by a liquid-nitrogen-cooled InSb photodetector. For each bias condition, we record the spectrally integrated EL signal $I_{R}$, EL (background-subtracted and integrated over the full EL peak) from the InSb detector.

The overall throughput of the collection optics and detection chain is calibrated using a quantum cascade laser (QCL) and a Lambertian reflectance standard (Thorlabs DG10-220-P01). A QCL emitting at a reference wavelength $\lambda_{\mathrm{ref}}$ is focused onto a diffuse reflector, which approximates a Lambertian source. The QCL output power at the sample plane, $P_{\mathrm{ref}}$, is first measured by a calibrated thermal power sensor placed at the focal plane of the reflective objective. Under identical optical alignment, the QCL light reflected from the Lambertian standard is then directed through the full collection optics and monochromator to the InSb detector, and the corresponding spectrally integrated signal $I_{R, \mathrm{ref}}$ is recorded. Because the QCL output can exceed the detector’s linear range, we placed a calibrated attenuator stack directly in front of the LN2-cooled InSb detector during QCL calibration, reducing the detected QCL signal to the same level as in EL measurements. The total transmission at $\lambda_{\mathrm{ref}}$ is denoted as T. With the attenuator stack in place during calibration, the ratio ${(P}_{\mathrm{ref}}$×T)/$I_{R,ref}$ provides the conversion factor from detector signal to optical power at $\lambda_{\mathrm{ref}}$ for our specific collection geometry.

For each EL measurement, the spectrally integrated EL signal $I_{R,EL}$ was obtained by background subtraction and integration over the full EL peak. The emitted optical power was then determined as

$$P_{\mathrm{EL}}=P_{\mathrm{ref}}\times T\times\left( I_{R,EL}/I_{R,ref} \right)\times\left[ \eta_{opt}\left( \lambda_{ref} \right)/\eta_{opt}\left( \lambda_{EL} \right) \right]\times\left[ D\left( \lambda_{ref} \right)/D\left( \lambda_{EL} \right) \right]$$

where $\eta_{opt}\left( \lambda\right)$ is the wavelength-dependent optical throughput of the collection path and monochromator, and $D\left( \lambda\right)$ is the wavelength-dependent responsivity of the InSb detector. $I_{R,EL}$ is the spectrally integrated EL signal for a given ($V_{\mathrm{ds}},V_{g}$) bias condition, and $I_{R,ref}$is the integrated detector signal from the QCL-illuminated Lambertian standard. *λ*_EL_ denotes the EL peak wavelength. The emitted photon flux is then

$$\Phi_{\mathrm{ph}}=P_{\mathrm{EL}}/E_{\mathrm{EL}}=P_{\mathrm{EL}}/\left( \mathrm{hc}/\lambda_{\mathrm{EL}} \right)$$

where $E_{\mathrm{EL}}$ is the photon energy corresponding to $\lambda_{\mathrm{EL}}$, h is Planck’s constant, and c is the speed of light. Combining the above expressions yields the working formula used to extract the EQE from the measured EL signal and drive current:

$$\eta_{\mathrm{EQE}}=\Phi_{\mathrm{ph}}/\left( I_{\mathrm{ds}}/q \right)=(qP_{\mathrm{EL}} )/\left( I_{\mathrm{ds}}E_{\mathrm{EL}} \right)$$

All EQE values were calculated at each measured bias point using the corresponding $I_{R,EL}$ and $I_{\mathrm{ds}}$ recorded from the $V_{\mathrm{ds}}$-sweep (fixed $V_{g}$) and $V_{g}$-sweep (fixed $V_{\mathrm{ds}}$) datasets.

The main remaining uncertainty in the EQE extraction is systematic. In particular, the QCL-illuminated diffuse reflector only approximates a Lambertian reference source. Any residual specular (mirror-like) reflection from the diffuse reflector could enhance the collected reference signal *I*_R, ref_ compared with a perfectly diffuse source. This would lead to an underestimation of the extracted EL power and hence of the calculated EQE. Accordingly, the reported EQE is likely a conservative lower bound for the true EQE.

**Supplementary Note 6.** **Spatially resolved EL mapping and 1064-nm reflection imaging**

To identify the spatial distribution of mid-infrared EL emerging from the Te/MoS_2_ heterojunction, we performed spatial mapping of the EL signal and compared it with a 1064-nm reflection map acquired over the same field of view. The sample was then raster-scanned by a motorized XY stage (step 1 $\mu m$) while the collection optics were fixed. The output of the detector at each position was recorded after subtracting the background signal.

To enhance the spatial selectivity of the EL mapping, a circular aperture (0.85 mm diameter) was positioned at the center of the 1064-nm reflection spot in the collection path to define a spatially restricted detection region. Because the reflective objective is infinity-corrected, the beam between the objective and downstream optics is approximately collimated. In this configuration, the pinhole functions as an angular stop, defining an effective detection area on the sample. Under the paraxial approximation, its acceptance half-angle is $\theta_{\max}\approx r/D$, where *r* is the pinhole radius and *D* is the objective-to-pinhole distance along the collimated beam. The corresponding effective detection diameter $d_{\text{eff}}$ on the sample can be estimated as:

$$d_{\text{eff}}\approx{2f}_{\text{obj}}\theta_{\max}\approx2f_{\text{obj}}\frac{r}{D},$$

where $f_{\text{obj}}$ is the effective focal length of the objective. Using *r*$=0.425 \text{mm, }D\approx580 \text{mm}$ and $f_{\text{obj}}\approx5.0 \text{mm}$ for the LMM40X-P01 objective, we obtain $d_{\text{eff}}\approx7.3 \mu m$, which corresponds to the spatial resolution of the EL measurement.

We first acquired a 1064-nm reflection map to identify the device outline and metal contacts and to provide an unambiguous geometric reference under identical alignment. Without changing the optical alignment or scan coordinates, we subsequently recorded the mid-infrared EL map over the same area. For EL mapping, the emission was collected by the same reflective microscope objective ($\times40$), spectrally filtered by a 3.5-µm bandpass window (3500 ± 500 nm), and detected by an InSb detector. A reflective objective lens is used to avoid chromatic dispersion between the 1064 nm reflection wavelength and the 3.5 µm emission wavelength.


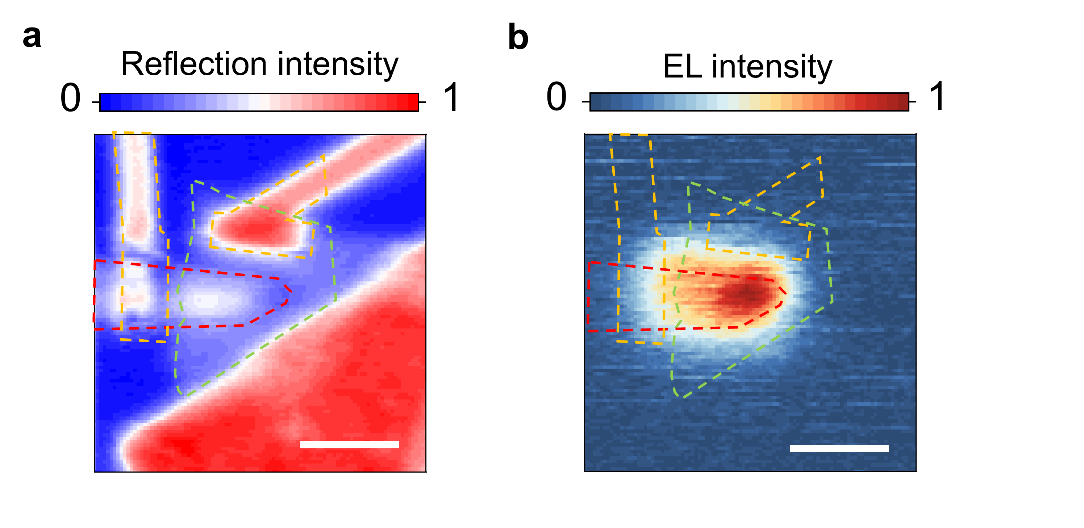


Fig. S5. Spatial mapping of mid-infrared EL and 1064-nm reflection maps. **a,** 1064-nm reflection map acquired over the same field of view to identify the device outline and metal contacts. The green dashed outline marks MoS_2_, the red dashed outline marks Te, and the yellow outline marks the metal electrodes. Scale bar: 30 μm. **b,** Integrated mid-infrared EL intensity map (3.0–4.0 µm bandpass) recorded under forward bias $\left( V_{g}=20 V,V_{\mathrm{ds}}=4 V;25 K \right).$ The EL map is compared with the 1064-nm reflection map in panel a. Scale bar: 30 μm.

**Supplementary Note 7.** **Measurement sensitivity in determining** $\boldsymbol{V}_{\mathbf{o}\mathbf{n}}$

In this Note, we clarify why the apparent EL turn-on voltage $V_{\mathrm{on}}$ depends on the detection scheme. In the spectrally resolved measurement (Fig. 3b), the EL signal is collected through a monochromator, where the finite optical throughput and slit-limited signal-to-noise ratio reduce the detected photon flux and effectively raise the detection threshold. In contrast, in the high-sensitivity configuration (Fig. 3c), the integrated mid-infrared EL intensity is directly recorded by an InSb photodetector through a 3–4 µm bandpass filter, avoiding wavelength-selection losses and enabling earlier detection of weak emission. This integrated detection yields an apparent $V_{\mathrm{on}}\approx1.7 \text{V}$, lower than the onset inferred from spectrally resolved data. Similar sensitivity-dependent shifts of the apparent turn-on have been noted when EL is measured with more sensitive photodiode-based detection.

This discrepancy is expected because $V_{\text{on}}$, as defined in the main text, is the minimum bias at which the integrated EL signal clearly exceeds the background floor; it is therefore an operational metric that depends on measurement sensitivity and detection bandwidth. A lower apparent $V_{\text{on}}$ correspondingly reduces the electrical input required to reach detectable emission, which is beneficial for lowering power consumption at turn-on.

**Supplementary Note 8. Polarization comparison in Te/TMD heterojunctions and isolated Te flakes**

Fig. S6 compares the normalized EL and PL degree of linear polarization (DOP) of two representative Te/TMD heterojunction LEDs measured at 25 K, including a Te/MoS_2_ device (red) and a Te/WSe_2_ device (blue). Fig. S6a shows the EL DOP, and Fig. S6b shows the PL DOP. While the drain bias and gate voltage conditions are not the same due to device variation on the turn-on behavior and the accessible operating windows are device dependent, we chose operating points with injection current densities that are as close as possible to enable a meaningful comparison. The DOP is normalized to the maximum value obtained for each device to highlight the relative polarization level.

For the Te/MoS_2_ device, the DOP was measured at an injection current density of approximately 0.85 μA μm^-2^ (using an effective emission area of about 300 μm^2^ to estimate the current density). For the Te/WSe_2_ device, the DOP was measured at an injection current density of approximately 0.88 μA μm^-2^ with an effective area of ~440 μm^2^. At these representative operating points, the corresponding absolute DOP values are ~0.70 for Te/MoS_2_ and 0.98 for Te/WSe_2_ (Fig. S6a).

To further examine whether the polarization variation is also reflected in optical excitation, we performed polarization-resolved micro-PL measurements on the corresponding Te flakes using an excitation power of 10 mW. The Te/WSe_2_ device shows a nearly unity PL DOP of ~0.98. In contrast, the Te/MoS_2_ device exhibits a much lower PL DOP of ~0.55 under our measurement conditions (Fig. S6b). Consistent with the discussion in the main text, Fig. S6 illustrates that different Te flakes can exhibit distinct EL/PL polarization levels. A plausible interpretation is that differences in the intrinsic doping level among Te flakes may lead to variations in carrier distribution during electrical injection, which could contribute to the observed device-to-device variation in DOP. In addition, local strain and substrate-induced effects are also possible contributing factors. Angle-resolved Raman spectroscopy was also performed to confirm the Te crystal orientation, as shown in Fig. S6c, where the crystal orientations are labeled with black arrows.


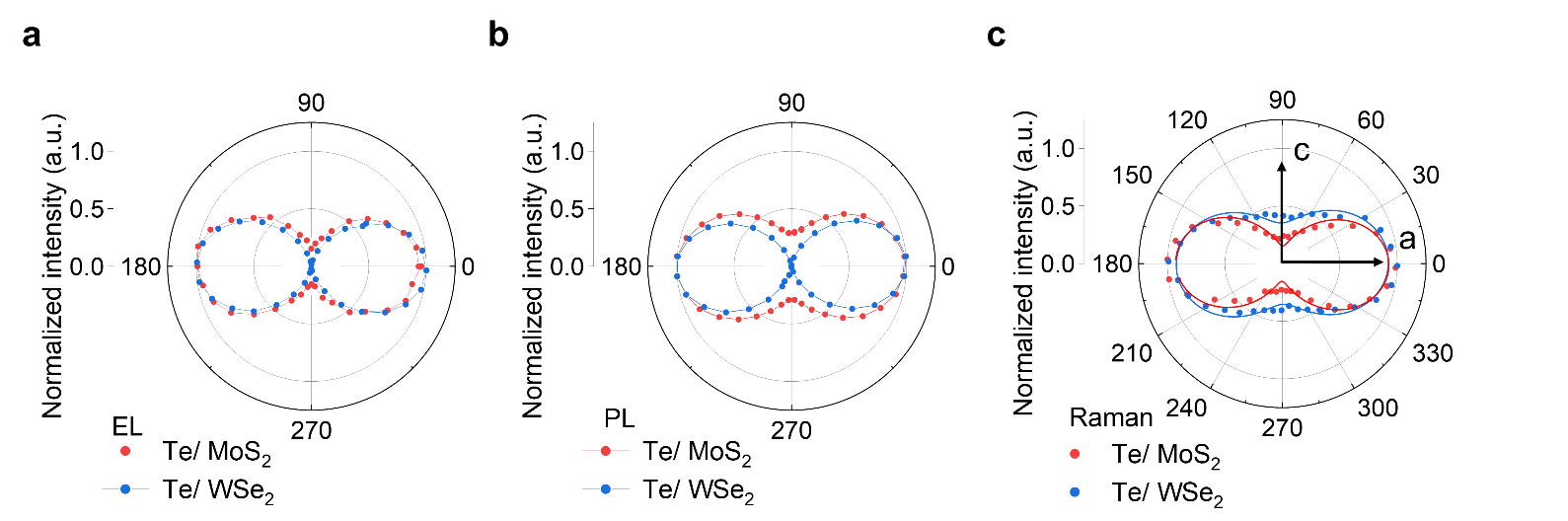


Fig. S6. **Comparison of polarization characteristics for two Te/TMD heterojunction emitters measured at 25 K.** Red symbols denote a Te/MoS_2_ device and blue symbols denote a Te/WSe_2_ device. The polarization values are normalized to each device’s maximum DOP to emphasize comparative trends. (a) Normalized EL DOP measured at matched injection conditions, with current density of ~0.85 μA μm^-2^ (~300 μm^2^) for Te/MoS_2_ and ~0.88 μA μm^-2^ (~440 μm^2^) for Te/WSe_2_. (b) Normalized PL DOP measured on the corresponding Te/TMD heterojunction by polarization-resolved micro-PL at 25 K using an excitation power of 10 mW. (c) Angle-resolved Raman polar plot of the A_1_ mode, used to determine the Te crystal orientation. The crystal orientations are labeled with black arrows.

To further verify that this variation is associated with the Te flakes themselves rather than the Te/TMD stacking geometry, we measured polarization-resolved PL from six isolated Te flakes without MoS_2_ or WSe_2_. As shown in Fig. S7, the normalized PL polar plots already show pronounced flake-to-flake variation. The first three Te flakes exhibit nearly unity DOP, whereas the other three show much lower DOP. Because no TMD layer is involved in these measurements, it reflects flake-dependent properties of Te itself.


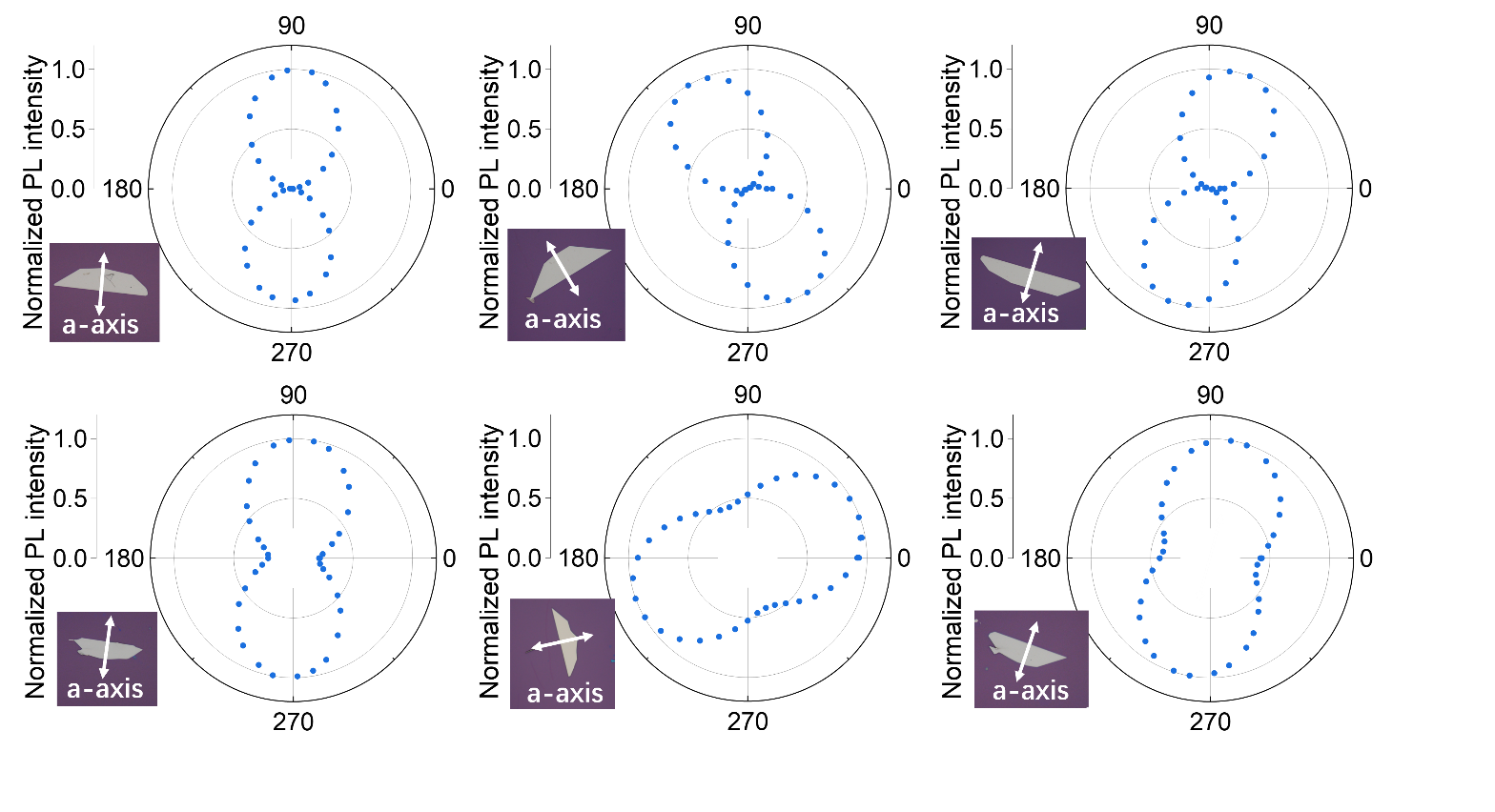


Fig. S7. **Polarization-resolved PL of isolated Te flakes at 25 K.** Normalized polarization-resolved PL polar plots measured from six individual Te flakes. The optical micrograph inset in each panel shows the corresponding Te flake and the a-axis direction.

**Supplementary Note 9.** **Long-term stability of electroluminescence**

To assess the long-term stability of our Te/MoS_2_ electroluminescent devices, we re-measured one representative device after prolonged storage and compared its EL characteristics with those obtained shortly after fabrication. The device was stored in an ambient condition for ~10 months and then re-characterized at 25 K using the same optical collection path and electrical measurement configuration. As shown in Fig. S8a, the maximum integrated EL intensity decreases from 1554 to 1415 after 10-month storage, corresponding to ~91% retention of the initial value. The EL response and turn-on behavior remain essentially unchanged within experimental uncertainty. Fig. S8b shows the normalized polarization-resolved EL before and after storage. The polarization axis remains unchanged, whereas the DOP changes slightly from ~0.66$\pm$0.01 to ~0.61$\pm$0.01. The DOP values were obtained by fitting the polarization-angle-dependent EL intensity with a cosine function and then extracting $DOP=(I_{max}-I_{min})/(I_{max}+I_{min})$. These results indicate that the Te/MoS_2_ heterojunction maintains robust EL performance after long-term storage, supporting the practical stability of the present device platform.


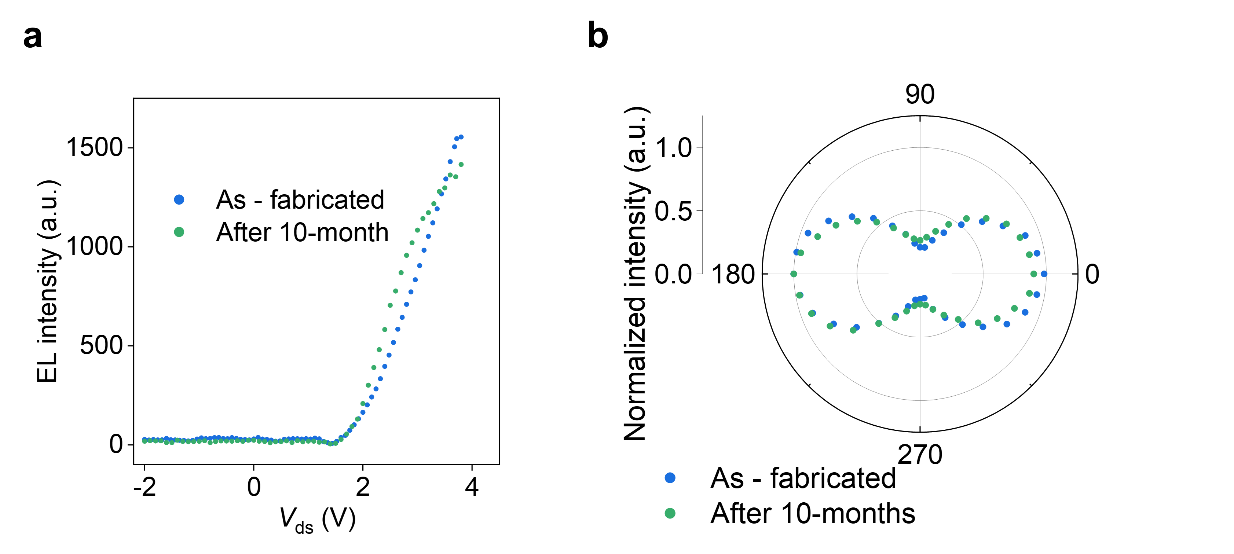


Fig. S8. Long-term stability. **a,** $V_{\mathrm{ds}}$-dependent integrated EL intensity measured at 25 K immediately after fabrication (blue) and 10 months later (green). The device was stored in ambient conditions. **b,** Normalized polarization-resolved EL of the same device before and after storage.

**Supplementary Note 10. Device‑to‑device reproducibility of gate‑modulated electroluminescence**

In order to assess the reproducibility of gate‑modulated mid‑infrared electroluminescence in Te/TMD heterojunction LEDs, we fabricated and measured multiple devices based on similar Te/TMD architectures. Representative optical micrographs and gate‑dependent EL responses of two independent devices are presented here to demonstrate that the observed emission behavior is not unique to a single sample, but is representative of the broader Te‑based heterojunction system. In each optical image, the Te flake region is outlined by a white dashed line, the MoS_2_ or WSe_2_ flake is outlined by green dashed line, and the metal electrodes (Pd/Au or Cr/Au) are outlined by yellow dashed lines.

Fig. S9a and S9b show measurements from a second Te/MoS_2_ heterojunction LED. The optical micrograph in Fig. S9a shows the device geometry including the Te and MoS_2_ flakes and the contact electrodes. Fig. S9b plots the integrated mid‑infrared EL intensity as a function of back‑gate voltage measured at a fixed drain bias of 6 V under identical optical collection conditions. The EL intensity exhibits a clear rise and fall behavior with increasing *V*_g_, qualitatively consistent with the gate‑modulated EL response presented in the main text for the first Te/MoS_2_ device. While the absolute intensity and the *V*_g_ window for peak emission vary somewhat due to differences in flake geometry and contact configuration, the overall trend of gate‑controlled EL remains consistent.

Fig. S9c and S9d show measurements from an independent Te/WSe_2_ heterojunction LED. The optical micrograph in Fig. S9c identifies the overlap region between the Te and WSe_2_ flakes, as well as the contact electrodes. The corresponding integrated EL intensity versus *V*_g_ data in Fig. S9d again display a gate‑modulated response under the same drain bias of 6 V. The overall shape of the EL-*V*_g_ curve is similar to those of the Te/MoS_2_ devices, indicating that gate‑controlled injection physics can be realized in Te/WSe_2_ heterostructures as well.

These additional device results provide evidence that the gate‑controlled injection physics and the associated electroluminescence characteristics can be reproduced in more than one device implementation. The inclusion of optical images with clear flake boundaries and EL response curves for multiple devices in the SI follows common practice in the emerging 2D emitter literature to demonstrate qualitative reproducibility across independently fabricated devices.


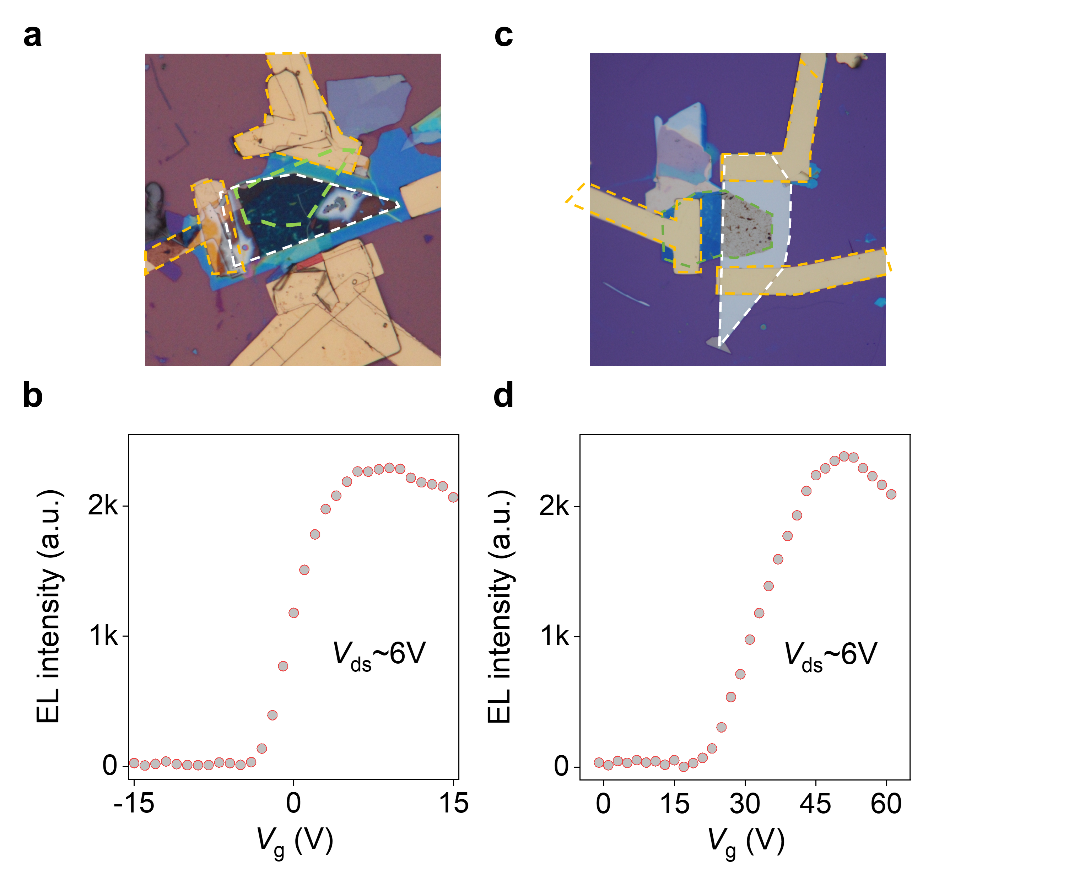


Fig. S9. **Gate‑voltage dependence of integrated EL intensity for multiple Te/TMD heterojunction LEDs measured at 25 K.** (a) Optical image of a Te/MoS_2_ device; (b) integrated EL vs *V*_g_ for the device in (a); (c) optical image of a Te/WSe_2_ device; (d) integrated EL vs *V*_g_ for the device in (c). Te flakes (white dashed), MoS_2_/WSe_2_ flakes (green dashed), and metal contacts (yellow dashed) are indicated.

**Supplementary Note 11.** **Gate‑Dependent polarization stability in electroluminescence**

In addition to the intensity modulation of electroluminescence (EL) under back‑gate control, we also examine the stability of the EL polarization state as a function of the back‑gate voltage. Fig. S10 presents the degree of linear polarization (DOP) measured at 25 K for a representative Te/MoS_2_ heterojunction LED under a fixed drain bias (*V*_ds_ ~3.5 V). Panel (a) compares the normalized polarization patterns measured at two representative back‑gate voltages (*V*_g_ = 10 V and *V*_g_ = 30 V). Panel (b) summarizes the extracted DOP values as a function of the back‑gate voltage from 5 V to 30 V, with error bars indicating the measurement uncertainty at each point.

The data show that, while the EL intensity can be strongly modulated by the back‑gate voltage, the DOP remains essentially constant (~0.58–0.59) over the gate sweep. This result demonstrates that the EL emission in this Te/MoS_2_ device exhibits polarization‑locked intensity control: the polarization state is not significantly altered by the back‑gate tuning that modulates injection efficiency. Together with the temperature‑dependent EL data presented elsewhere in the Supplementary Information, these results support a coherent picture in which the EL intensity can be tuned by electrostatic control without altering the polarization state that is intrinsic to the Te flake and device configuration.


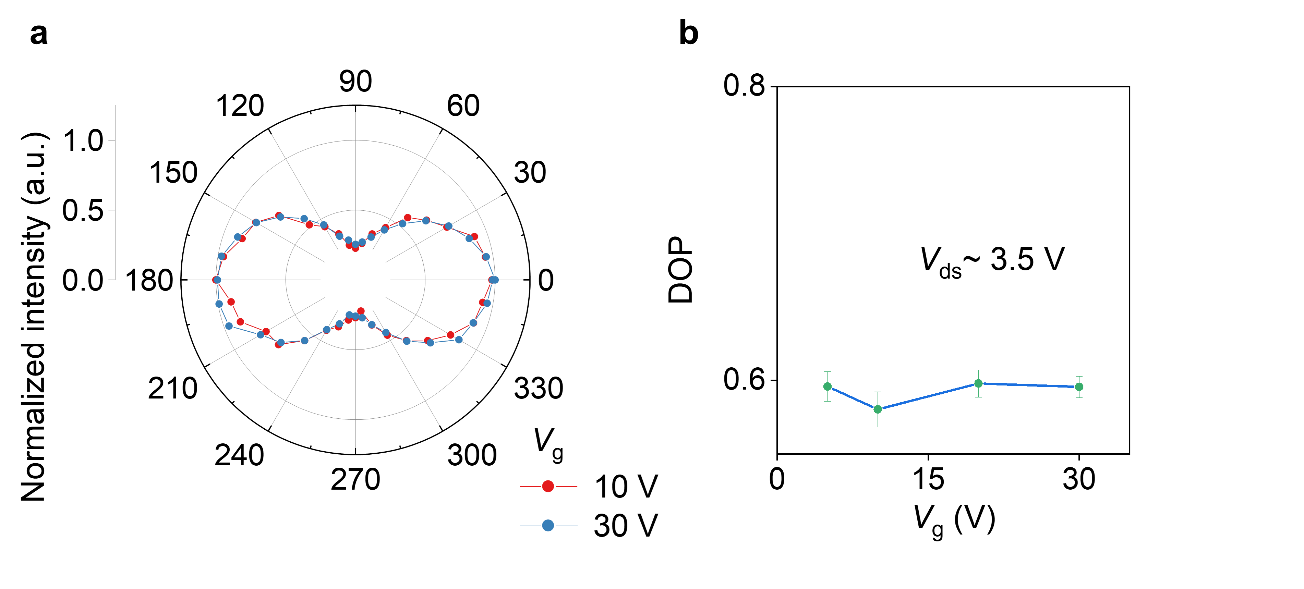


**Fig. S10. Gate‑voltage dependence of the degree of linear polarization (DOP) in a Te/MoS_2_ heterojunction LED measured at 25 K with fixed drain bias (**$\boldsymbol{V}_{\mathbf{ds}}$ **~3.5 V).** (a) Normalized polarization patterns measured at *V*_g_ = 10 V (red) and *V*_g_ = 30 V (blue); (b) Extracted DOP as a function of back‑gate voltage from 5 V to 30 V, with error bars indicating measurement uncertainty.

**Supplementary Note 12. Linewidth analysis of bias- and gate-dependent electroluminescence**

We extracted the full width at half maximum from all EL spectra using the same half-maximum method. The error bars in Fig. S11 represent the combined uncertainty from statistical errors and noise-induced errors. As shown in Fig. S11a, for the bias-dependent EL spectra measured at fixed *V*_g_ = 20 V, the full width at half maximum decreases from about 17 meV at *V*_ds_ = 3.0 V to about 12 meV at *V*_ds_ = 4.0 V, indicating a weak linewidth narrowing trend with increasing forward bias. However, the magnitude of this change remains relatively small compared with the absolute linewidth itself. This narrowing trend does not mainly originate from a pronounced shift of the main emission peak. Instead, it is primarily associated with the reduced relative contribution from the low-energy side of the asymmetric EL spectrum. To quantify this asymmetry, we further decompose the linewidth as FWHM = FWHM_l_ + FWHM_h_, where FWHM_l_ (FWHM_h_) denotes the energy width from the peak position to the half-maximum point on the low-energy (high-energy) side. As shown in Fig. S11a, FWHM_l_ decreases noticeably with increasing *V*_ds_ (or emission intensity), whereas FWHM_h_ remains relatively unchanged. At low bias and under weak emission conditions, the low-energy-side intensity makes a noticeable contribution, which shifts the left half-maximum point toward lower energy and results in a larger apparent full width at half maximum. With increasing *V*_ds_, the emission becomes increasingly dominated by band-edge recombination, while the relative contribution from the low-energy-side emission decreases. The low-energy in-gap emission may originate from shallow impurity states, the Franz-Keldysh effect induced by the built-in electric field, and excitonic emission. Under stronger excitation, these contributions gradually become saturated or are masked by the stronger band-edge emission.

To evaluate the effect of gate control on the EL linewidth, we performed the same linewidth analysis on the gate-dependent EL spectra shown in Fig. 4d. As shown in Fig. S11b, for the gate-dependent EL spectra measured at a fixed forward-bias condition of ~4 V, the full width at half maximum first decreases from about 13 meV at *V*_g_ = 5 V to about 10 meV at *V*_g_ = 20 V, where the EL emission is strongest, and then slightly increases to about 12 meV at *V*_g_ = 30 V. This overall variation remains small compared with the absolute linewidth itself. Notably, a narrower linewidth is correlated with stronger EL emission. This trend is consistent with the bias-dependent behavior in Fig. S11a. Therefore, the weak linewidth evolution with gate voltage is more naturally attributed to changes in the relative contribution of the low-energy-side emission under different injection conditions.


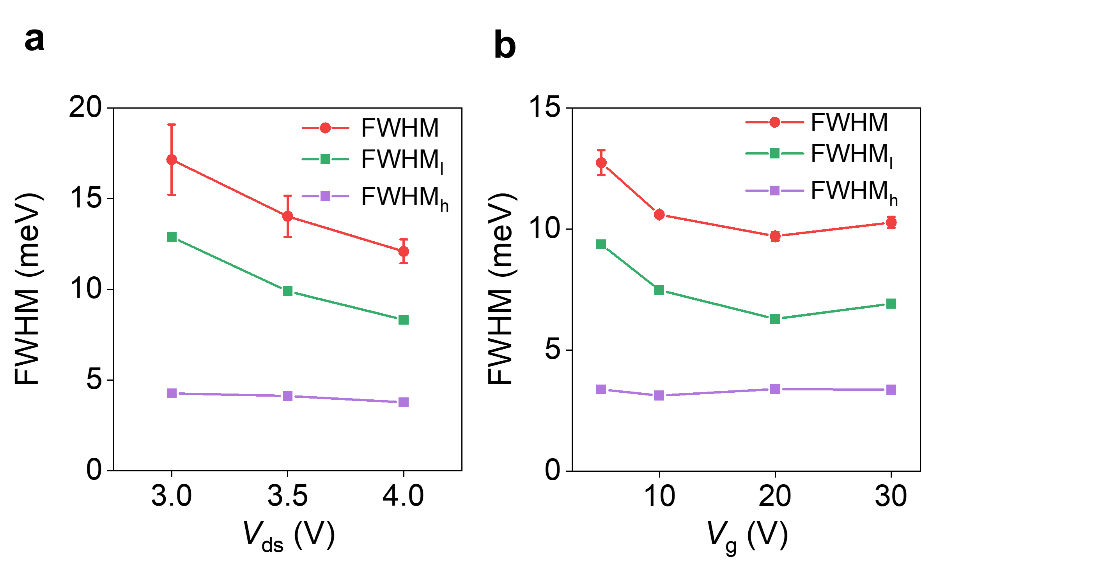


**Fig. S11. Extracted EL full width at half maximum under bias and gate modulation.** (a) FWHM of the bias-dependent EL spectra measured at *V*_ds_ = 3.0, 3.5, and 4.0 V, with *V*_g_ fixed at 20 V. (b) FWHM of the EL spectra measured under different back-gate voltages at a fixed forward-bias ~4 V. FWHM (red), FWHM_l_ (green), and FWHM_h_ (purple) are plotted, where FWHM_l_ and FWHM_h_ denote the half widths from the peak energy to the half-maximum points on the low-energy and high-energy sides, respectively. The error bars represent the combined uncertainty from statistical errors and noise-induced errors.
